# Chicken bone marrow mesenchymal stem cells improve lung and distal organ injury

**DOI:** 10.1038/s41598-021-97383-4

**Published:** 2021-09-10

**Authors:** Hexuan Niu, Hanan Song, Yuhan Guan, Xianchun Zong, Ruili Niu, Shiyu Zhao, Cong Liu, Wenzaixiang Yan, Weijun Guan, Xishuai Wang

**Affiliations:** 1 Department of Animal Genetic Resources, Institute of Animal Science, Chinese Academy of Agricultural Sciences, 2 Yuanmingyuan Road, Beijing, 100193 People’s Republic of China; 2 College of P.E and Sport, Beijing Normal University, Beijing, 100875 People’s Republic of China; 3 Harbin Sport University, Harbin, 150008 People’s Republic of China; 4 The University of British Columbia, 2329 West Mall, Vancouver, BC Canada; 5 College of Life Science and Technology, Mudanjiang Normal University, Mudanjiang, 157011 People’s Republic of China

**Keywords:** Cell migration, Cell signalling, Chemokines, Infection, Infectious diseases, Inflammation, Neuroimmunology

## Abstract

Mesenchymal stem cells (MSCs) are associated with pulmonary protection and longevity. We separated chicken bone marrow-derived mesenchymal stem cells (BM-MSCs); investigated whether BM-MSCs can improve lipopolysaccharide (LPS)-induced lung and distal organ injury; and explored the underlying mechanisms. Ninety-six male ICR (6 weeks old) mice were randomly divided into three groups: Sham, LPS, and LPS + MSC groups. The mice were intratracheally injected with 5 mg/kg LPS to induce acute lung injury (ALI). The histopathological severity of injury to the lung, liver, kidney, heart, and aortic tissues was detected. Wet/dry ratio, protein concentrations in bronchoalveolar lavage fluid (BALF), BALF cell counts, inflammatory cytokine levels in serum, inflammatory cytokine gene expression, and oxidative stress-related indicators were detected. In addition, a survival analysis was performed in sixty male ICR mice (6 weeks old, 18–20 g). This study used chicken BM-MSCs, which are easier to obtain and more convenient than other animal or human MSCs, and have MSC-associated properties, such as a colony forming ability, multilineage differentiation potential, and certain phenotypes. BM-MSCs administration significantly improved the survival rate, systemic inflammation, and the histopathological severity of lung, liver, kidney, and aortic injury during ALI. BM-MSCs administration reduced the levels of inflammatory factors in BALF, the infiltration of neutrophils, and oxidative stress injury in lung tissue. In addition, BM-MSCs administration reduced TRL4 and Mdy88 mRNA expression during ALI. Chicken BM-MSCs serve as a potential alternative resource for stem cell therapy and exert a prominent effect on LPS-induced ALI and extrapulmonary injury, in part through TRL4/Mdy88 signaling and inhibition of neutrophil inflammation and oxidative stress injury.

## Introduction

Acute lung injury (ALI), which is characterized by increased lung permeability and pulmonary inflammation, leads to diffuse alveolar damage, lung edema, hypoxia, and respiratory failure and exhibits high mortality rates^1–3^.

The pathogenesis of ALI is complex, and excessive inflammation plays a critical role^4,5^. ALI is characterized by an uncontrolled inflammatory response involving inflammatory mediators, including IL-1β and TNF-α, and effector cells, among which neutrophils play a key role^6,7^. Neutrophils release multiple factors, such as profibrogenic factors matrix metalloproteinase 9 (MMP-9) and transforming growth factor-beta (TGF-β)^8–11^. Hence, ALI may be accompanied by pulmonary fibrosis. Deleterious activation of neutrophils led to collateral tissue damage^12^. Therefore, ALI may be accompanied by extrapulmonary injuries, such as liver, kidney, heart, and aortic injury. Previous studies have demonstrated that ALI causes acute organ dysfunction, including dysfunction of the cardiovascular system^13^, which delivers oxygen, nutrients, inflammatory cells, and inflammatory cytokines to tissues and seriously impairs organ function, but previous studies have ignored the effects of mesenchymal stem cells (MSCs) on the cardiovascular system in ALI models. Previous researches reported that the inflammatory activation of the human artery endothelial cells required Toll-like receptor 4 (TLR4) to induce the pro-inflammation factors, such as TNF-α and IL-1β^14,15^. Activation of TLR4/myeloid differentiation factor (88MyD88) signaling has been confirmed to lead to a variety of diseases, including ALI^16^. In mice with ALI, LPS led to an excessive inflammatory response via TLR4/MyD88 signaling, but the effects of BM-MSCs on TLR4/MyD88 signaling are unclear.

MSCs, which have a typical fibroblast-like morphology, enormous proliferative potential, and multilineage differentiation potential, are used to treat many autoimmune and inflammatory diseases, including ALI and sepsis, due to their low immunogenicity and potent immunomodulatory and paracrine signaling capacities^17,18^. Increasing evidence has demonstrated that MSC administration either systemically or locally at concentrations ranging from 0.5 × 10^6^ to 200 × 10^6^ cells can achieve positive outcomes in mice with ALI^19–21^.

Humans have studied eggs for 2500 years. The egg is still the best model organism in science, and it is also the medium used by humans to make immune serum every year. The main source of flu vaccine ingredients used by people is chickens. Human proteins can be used to produce antibodies to fight diseases, but the cost is high, and the process is complicated. Eggs are very cheap and suitable for mass production. Unlike other species, such as sheep or hamsters, chickens produce protein in a surprisingly similar way to humans. So-called genetically modified chickens can be obtained by implanting genes from other species, including human genes, into the body of chickens. Chicken BM-MSCs offer multiple applicative advantages. MSCs can easily be isolated, cultured, and propagated in vitro. Compared with embryonic stem cells (ESCs), MSCs have a limited risk of tumor formation and do not pose an ethical controversy^21^. However, the vast majority of studies on MSCs mainly concentrate on cells from humans, mice, and rats, but numerous sources for producing these MSCs are not available, and the MSCs cannot be harvested at a low cost. Compared with the species mentioned above, chicken embryos are widely available and are generally the cheapest choice at present. More importantly, the time needed for egg incubation is only 21 days; hence, tissues can be conveniently collected at specific stages of development. The timing of sampling exerts a substantial effect on the activity and proliferative capacity of MSCs^22^. Generally, if the timing of sampling is too early, tissues are not yet formed; if the timing of sampling is too late, the viability of the MSCs is significantly decreased. Hence, this study could reduce the cost of MSC research and contribute to further studies and the application of MSCs. We attempted to provide a scientific foundation for the use of chicken BM-MSCs and to identify whether BM-MSCs can reduce ALI and extrapulmonary injury.

We aimed to identify whether BM-MSCs can prevent ALI and distal organ injury. In addition, the underlying molecular mechanisms through which BM-MSCs modulated inflammatory parameters and the oxidative stress index in LPS-induced ALI have not yet been fully elucidated.

## Methods

### Isolation, culture, and cryopreservation of chicken BM-MSCs

Chicken bone marrow was obtained from 16-day-old chicken embryos. The connective tissues were carefully removed. Complete DMEM/F12 medium was utilized to flush the marrow cavity. Complete medium containing BM-MSCs was filtered through a 100-µm mesh sieve. After centrifugation, the cell pellets were resuspended in complete medium; seeded into 6-well plates (10^4^ cells per well); and cultured at 37 °C. At 48 h post-seeding, the supernatant was discarded, the nonadherent cells and exfoliated tissue masses were removed. Thereafter, the medium was replaced every 24–48 h. 0.25% trypsin/EDTA was utilized to passage the BM-MSCs at a 1:2 dilution.

Cryopreservation of MSCs was essential for further research. When the BM-MSCs reached 90% confluence, they were digested and harvested in 15-mL sterile centrifuge tubes. The cell pellets were resuspended in freezing medium composed of 50% FBS, 40% DMEM/F12, and 10% DMSO. One milliliter of the resulting suspension (1 × 10^6^ cells/mL) was stored at − 80 °C overnight, and then, the BM-MSCs were stored at liquid nitrogen tanks.

### Flow cytometry

Using an established procedure, BM-MSCs in the logarithmic phase were dissociated into a single-cell suspension. After counting, 1 × 10^6^ cells were transferred to a sterile centrifuge tube. Precooled 70% ethanol was added dropwise to fix the BM-MSCs, and then, the cells were gently prepared as a single-cell suspension, followed by incubation for 12 h. BM-MSCs were incubated with PBS containing rabbit-derived primary antibodies against CD73, CD90, CD105, OCT-4, SOX-2, CD34, and CD45. FITC-labeled goat anti-rabbit IgG was incubated with MSCs in the dark for 60 min. Finally, the MSCs were subjected to flow cytometry analysis.

### Identification of the multilineage differentiation potential of chicken BM-MSCs

#### Detection of osteogenic differentiation

The complete medium was replaced with osteogenic differentiation medium when BM-MSCs reached 30% confluence. Half of osteogenic differentiation medium was replaced with fresh osteogenic differentiation medium every other day. On days 21 of culture and differentiation, calcium deposition was identified via Alizarin Red staining. All information on the induction medium is listed in Supplementary Material 1.

#### Detection of adipogenic differentiation

Third-generation BM-MSCs were inoculated in 6-well plates. The medium was replaced with adipogenic induction and differentiation medium when cells were 30% confluent. Adipogenic induction and differentiation medium was refreshed every other day for 21 days. Afterward, Oil Red O staining was used to detect the accumulation of intracellular lipid droplets.

#### Detection of chondrogenic differentiation

For chondrogenic differentiation, third-generation BM-MSCs were cultured in chondrogenic medium when they proliferated to 30% confluence. On day 21 of culture, Alcian blue staining was performed to confirm chondrogenic differentiation.

#### Detection of neuroblastic differentiation

When 30% confluence was achieved, BM-MSCs were exposed to neuroblastic differentiation medium. On day 21 of differentiation and culture, cellular morphology was detected.

### LPS-induced ALI model

All protocols used in this study were approved by the Animal Experimental Welfare of the Institute of Animal Science, Chinese Academy of Agricultural Sciences (Beijing, China). All experiments were performed in accordance with the Animal Experimental Welfare of the Institute of Animal Science, Chinese Academy of Agricultural Sciences and the Guide for the Care and Use of Laboratory Animals published by the US National Institutes of Health. The authors have read the ARRIVE guidelines and the study was carried out in compliance with the ARRIVE guidelines. All mice were anesthetized via intraperitoneal injection of pentobarbital (0.2 mg/kg). The mice were euthanized with isoflurane.

The ALI model was established by administering an intratracheal injection of 5 mg/kg LPS (O55:B5, Sigma-Aldrich, St. Louis, MO, USA). Ninety-six mice were randomly assigned to one of three groups: (1) saline group (Sham), mice were intratracheally injected with a volume of normal saline equal to the volume of LPS administered in the other groups; (2) LPS group (LPS), mice were intratracheally injected with 5 mg/kg LPS; and (3) LPS + MSC group (LPS + MSC), mice were intratracheally injected with 5 mg/kg LPS. Two hours later, the mice received 1 × 10^6^ chicken BM-MSCs via tail vein injection. All mice were intraperitoneally injected with the anti-rejection drug cyclosporine once a day for one week before the experiment. The mice were euthanized at 6 h, 12 h, 24 h, and 48 h. Each group contained eight mice.

### Detection of the homing of BM-MSCs in lung tissue

To identify the homing of BM-MSCs in lung tissue, BM-MSCs were treated with CM-Dil (C7000, Life Technologies, Eugene, Oregon, USA) for 30 min in an incubator in the dark before BM-MSCs injection. Twenty-four mice were divided into three groups: (1) saline group (Sham), mice were intratracheally injected with a volume of normal saline equal to the volume of LPS; (2) LPS group (LPS), mice were intratracheally injected with 5 mg/kg LPS; and (3) LPS + MSCs group (LPS + MSC), mice were intratracheally injected with 5 mg/kg LPS. BM-MSCs were treated with CM-Dil for 30 min. An hour and a half later, 1 × 10^6^ CM-Dil-labeled BM-MSCs was injected in caudal vein. Two weeks later, mice were sacrificed. Frozen lung tissue sections were examined to observe CM-Dil-labeled BM-MSCs via fluorescence microscopy.

### Collection of BALF and tissue samples

We harvested blood samples of mice from the retro-orbital sinus. Blood samples were placed into blood collection vessels containing an anticoagulant. After centrifugation, the upper serum layer was frozen at − 20 °C. After sampling, the tissues were immediately transferred to liquid nitrogen tanks.

Two milliliters of ice-cold PBS was utilized to collect bronchoalveolar lavage fluid (BALF), the whole BALF was flushed three times, and the output fluid was harvested. The supernatant was immediately stored at − 20 °C.

### Histopathology

For the histological analysis, lung, liver, kidney, heart, and aortic tissue samples were dehydrated and embedded in paraffin. 5-μm-thick sections were stained with hematoxylin and eosin (HE) after administration of 5 mg/kg LPS for 6 h, 24 h, and 48 h. Following paraffin sectioning, 5-μm-thick sections were stained with Masson and Sirius Red after administration of 12 mg/kg LPS for 4 weeks. Images were captured with an inverted microscope and utilized to determine the degree of organ injury.

### Detection of pulmonary permeability

The wet–dry weight (W/D) ratio of lung tissue and the protein content in the BALF were detected to quantify the degree of pulmonary permeability. Bibulous papers were utilized to absorb liquid and blood from the surface of the lung tissue, and then, the wet weight of lung tissue was determined. The lung tissue was dried until a stable dry weight was obtained. The W/D ratio was calculated according to the following formula: W/D = wet weight of the left lower lobe/dry weight of the left lower lobe. A Bio-Rad protein assay kit was utilized to calculate the protein concentration in the BALF. Evans blue (Sigma, St. Louis, MO, USA) extravasation from the lung tissue was detected with a spectrophotometer.

### Determination of the oxidative stress index

MDA, SOD, MPO, and GSH levels in the mice were detected using spectrophotometry (Bio-Tek Instruments Inc., software KC4 v3.0), as previously described according to the manufacturer’s instructions.

### Evaluation of cytokines in serum

Based on the manufacturer’s specifications, the serum levels of CXCL-1, TNF-α, IL-1β, IL-8, and TNF-α were detected using mouse ELISA kits (Neobioscience).

### Evaluation of cytokines in BALF

Based on the manufacturer’s specifications, the CXCL-1, IL-1β, IL-1RN, IL-8, IL-10, TNF-α, MMP-9, and TGF-β levels in BALF were detected with mouse ELISA kits (Neobioscience).

### Measurement of extrapulmonary injury markers

For histological analysis, liver, kidney, heart, and aortic tissue samples were fixed in 4% PFA for 24 h and embedded in paraffin. Following the paraffin sectioning method, 5-mm-thick sections were stained with hematoxylin and eosin (HE). Images were captured with an inverted microscope.

According to the manufacturer’s specifications, the levels of markers of liver disease (ALT and AST) and kidney injury (urea and BUN) in serum were detected using commercial kits (Rsbio, Shanghai, China).

### Quantitative real-time PCR

Total RNA of lung tissues was extracted and used to synthesize cDNAs. Relative gene expression levels were measure using real-time fluorescence quantitative PCR. We selected glyceraldehyde-3-phosphate dehydrogenase (GAPDH) as the internal reference gene. Quantitative analysis was performed using the 2^−△△CT^ method. Supplementary Material 2 showed the murine PCR primer sequences.

### Assessment of the survival rate

For survival analyses, 60 mice were divided into the following groups: (1) saline group (Sham), in which the mice were intratracheally injected with the same volume of saline as the volume of LPS; (2) LPS group, in which the mice were intratracheally injected with 12 mg/kg LPS; and (3) LPS + MSCs group (LPS + MSC), in which the mice were intratracheally injected with 12 mg/kg LPS and then received 1 × 10^6^ chicken BM-MSCs via a tail vein injection two hours later. ALL mice were intraperitoneally injected with the anti-rejection drug cyclosporine for one week before the experiment. The number of dead mice was recorded every 6 h for 48 h. Each group included 20 mice. Four weeks later, all the mice were sacrificed. All tissue samples were fixed, dehydrated, and embedded in paraffin. 5-μm sections of lung tissues were got, deparaffinized, and stained with Masson and Sirius Red.

### Statistical analysis

We performed one-way ANOVA and graphed the figures using GraphPad Prism 9 software. The significance threshold was set to *P* < 0.05. All data are expressed as the mean ± SD (x ± s).

## Results

### Isolation and characterization of chicken BM-MSCs

**Figure 1:**
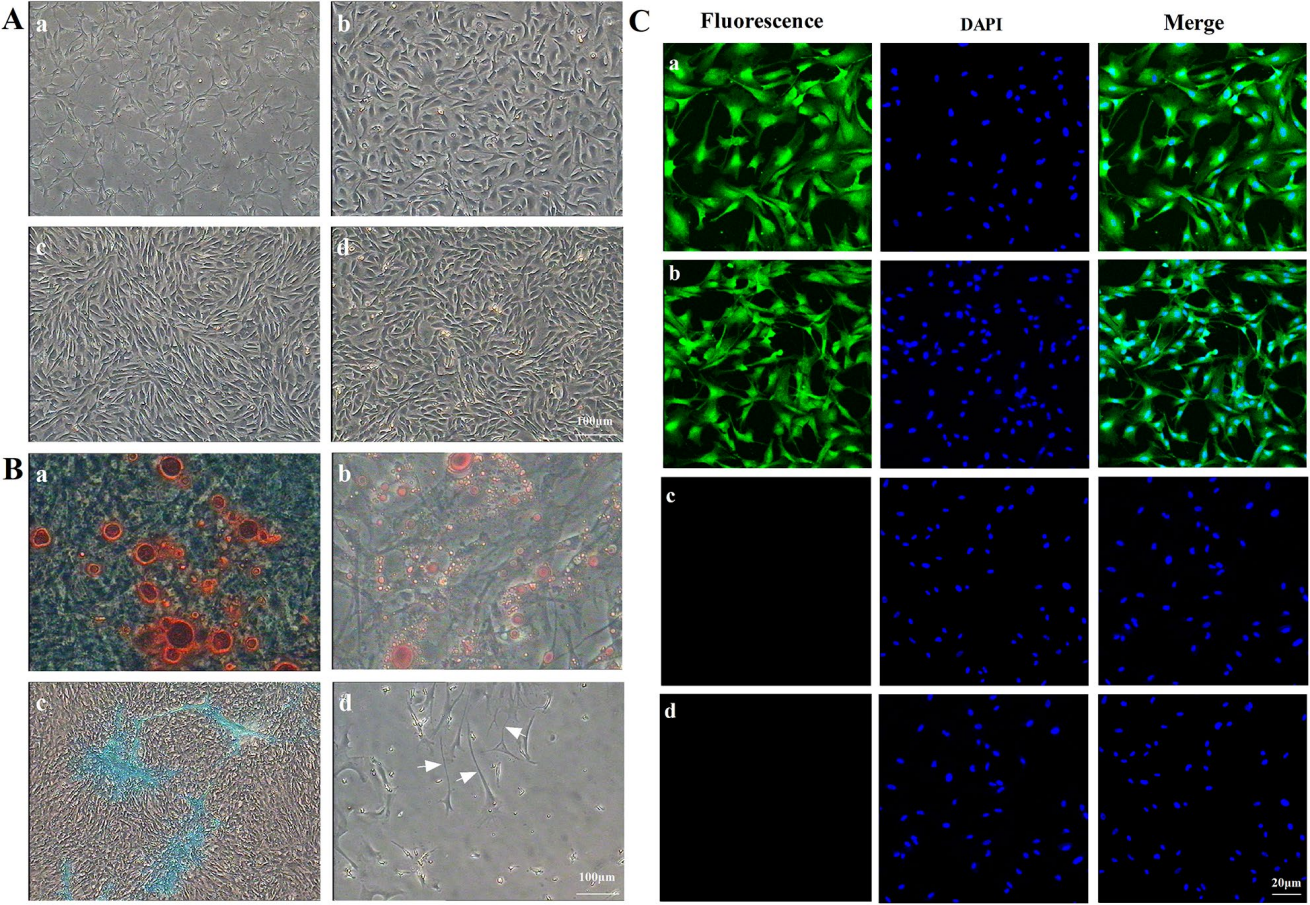
Biological characteristics of BM-MSCs. (**A**) The cellular morphology of chicken BM-MSCs was determined at different passages: P1 (a), P5 (b), P15 (c), and P25 (d). BM-MSCs were spindle-like cells. (**B**) Detection of the multilineage differentiation potential of BM-MSCs. Many calcium nodules appeared in the cytoplasm, and Alizarin red staining showed a positive reaction for calcified nodules in BM-MSCs that differentiated under osteogenic induction conditions for 21 days (a). Many lipid droplets formed in the cytoplasm of cells cultured under adipocytic induction conditions for 21 days and lipid droplets were successfully stained with Oil Red O (b). BM-MSCs were positive for Alcian blue staining after incubation with chondrogenic medium for 21 days (c). The cellular morphology changed from a spindle-shaped morphology to a multipolar and stellate morphology, and the BM-MSCs grew many branches and formed many synapses (white arrows) on the cell surface after culture under neurogenic induction conditions for 21 days (d). (**C**) The results of immunofluorescence staining. High expression of Nestin (a) and MAP-2 (b), markers of neural cells, was detected in differentiated BM-MSCs, while Nestin (c) and MAP-2 (d) were not detected in undifferentiated BM-MSCs. (**A** and **B**, ×40 magnification. **C**, ×200 magnification).

The cellular morphology of chicken BM-MSCs was determined at different passages (P1, P5, P15, and P25) (Fig. 1A, a–d). After culture under osteogenic induction conditions for 21 days, many calcium nodules appeared in the cytoplasm, and Alizarin red staining showed a positive reaction for calcified nodules in differentiated BM-MSCs (Fig. 1B-a). After culture under adipocytic induction conditions for 21 days, many lipid droplets formed in the cytoplasm, and the lipid droplets were successfully stained with Oil Red O (Fig. 1B-b). BM-MSCs displayed positive Alcian blue staining after incubation with chondrogenic medium for 21 days (Fig. 1B-c). Culture under neurogenic induction conditions for 21 days changed the cellular morphology from a spindle-shaped morphology to a multipolar and stellate morphology, and the BM-MSCs grew many branches and formed many synapses on the cell surface, as indicated by the white arrows (Fig. 1B-d). The results of immunofluorescence staining revealed the expression of the neural cell markers Nestin (Fig. 1C-a) and MAP-2 (Fig. 1C-b), while undifferentiated cells did not express Nestin (Fig. 1C-c) or MAP-2 (Fig. 1C-d). As shown in Supplementary Material 3, the BM-MSCs surface antigens CD73, CD90, CD105, Sox-2, and OCT-4 were positively expressed, while CD34 and CD45 were not expressed, as identified by flow cytometry.

### BM-MSCs administration reduced mortality

**Figure 2:**
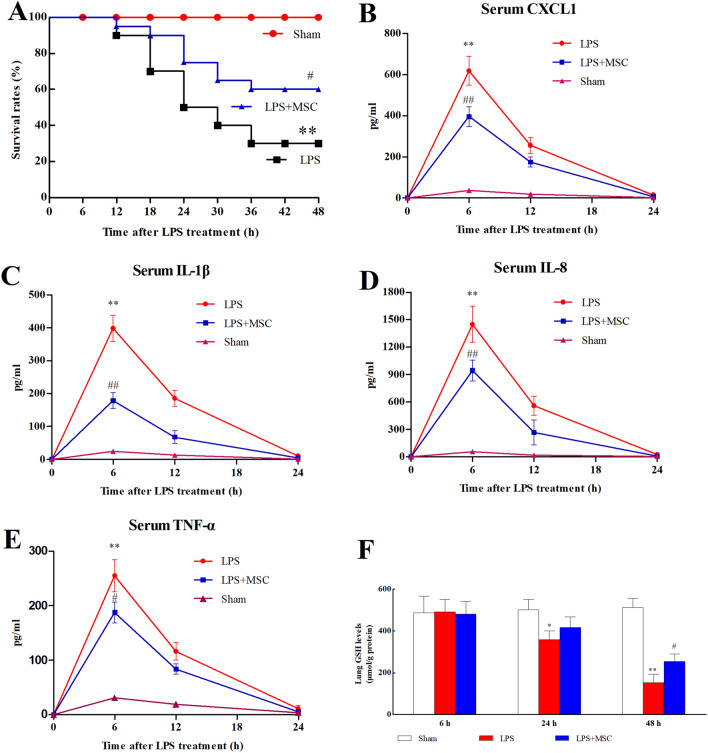

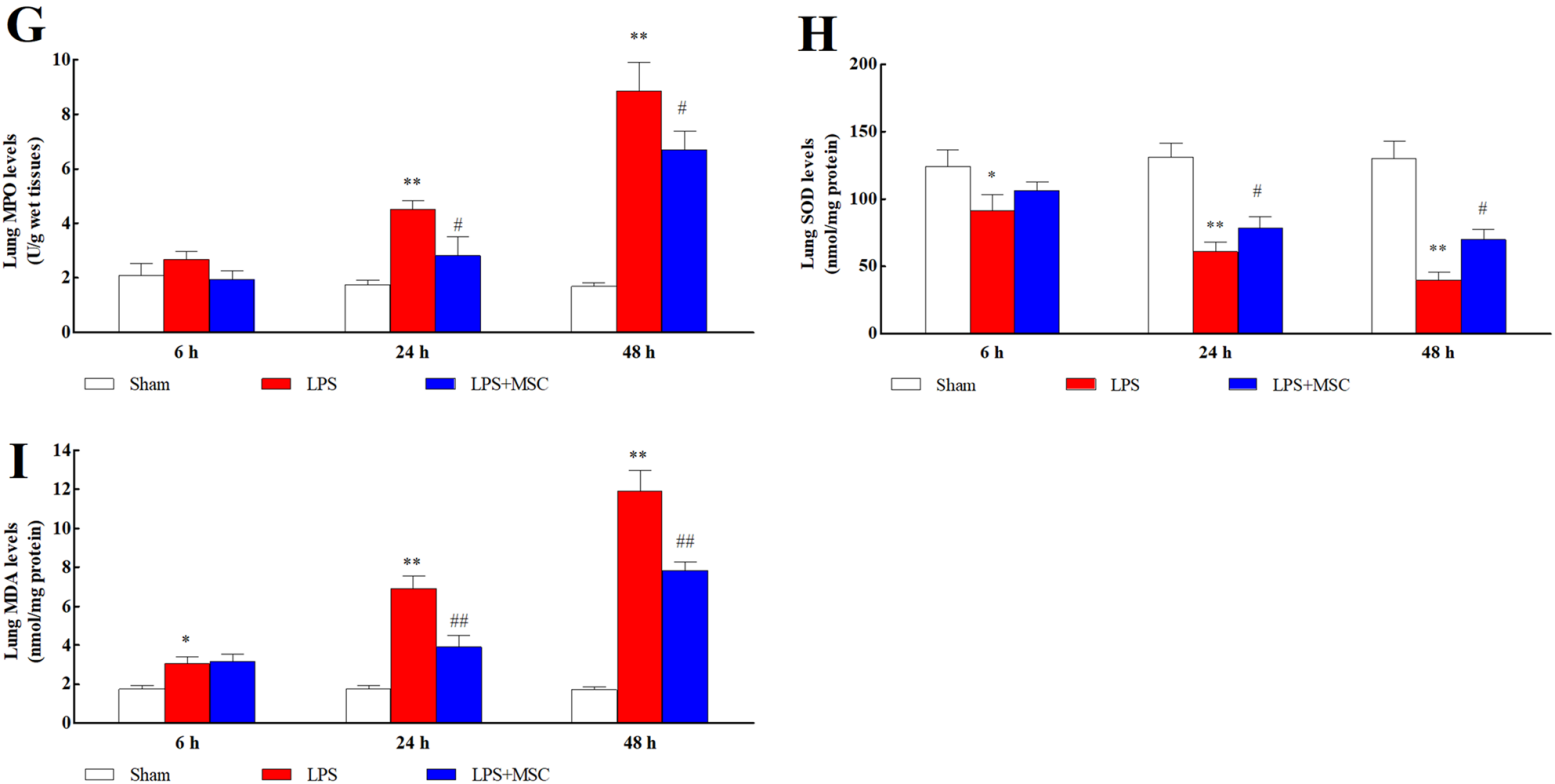
Assessment of survival rates, systemic inflammation, and oxidative stress injury. (**A**) Assessment of survival rates. The serum levels of CXCL-1 (**B**), IL-1β (**C**), IL-8 (**D**), and TNF-α (**E**) were detected. The levels of SOD (**F**), MDA (**G**), GSH (**H**), and MPO (**I**), as indicators of oxidative stress injury, were detected. *P < 0.05 versus the Sham group; ^#^*P* < 0.05 versus the LPS group; **P < 0.01 versus the Sham group; ^##^*P* < 0.01 versus the LPS group. Values are expressed as the mean ± SD.

All sham-operated mice survived. BM-MSCs treatment prominently improved the survival rate during ALI compared to LPS (Fig. 2A; *P* < 0.05).

### BM-MSCs administration reduced systemic inflammation

The serum levels of CXCL-1 (Fig. 2B; *P* < 0.05), IL-1β (Fig. 2C; *P* < 0.05), IL-8 (Fig. 2D; *P* < 0.05), and TNF-α (Fig. 2E; *P* < 0.05) were prominently increased within 6 h and decreased at 12 h and 24 h compared to Sham. The administration of BM-MSCs prominently prevented the increase in serum levels of IL-1β, IL-8, CXCL-1, and TNF-α compared to LPS (*P* < 0.05).

### BM-MSCs administration improved oxidative stress parameters

The expression of SOD (Fig. 2F), MDA (Fig. 2G), GSH (Fig. 2H), and MPO (Fig. 2I) in lung tissue were detected. LPS administration prominently increased the expression of MDA (*P* < 0.05) and MPO (*P* < 0.05) and prominently decreased the expression of SOD (*P* < 0.05) and GSH (*P* < 0.05) compared to Sham. Significant increases in the MDA (*P* < 0.05) and MPO (*P* < 0.05) levels and significant decreases in SOD (*P* < 0.05) and GSH (*P* < 0.05) levels were detected after BM-MSCs administration compared to LPS.

### BM-MSCs administration reduced lung injury

**Figure 3:**
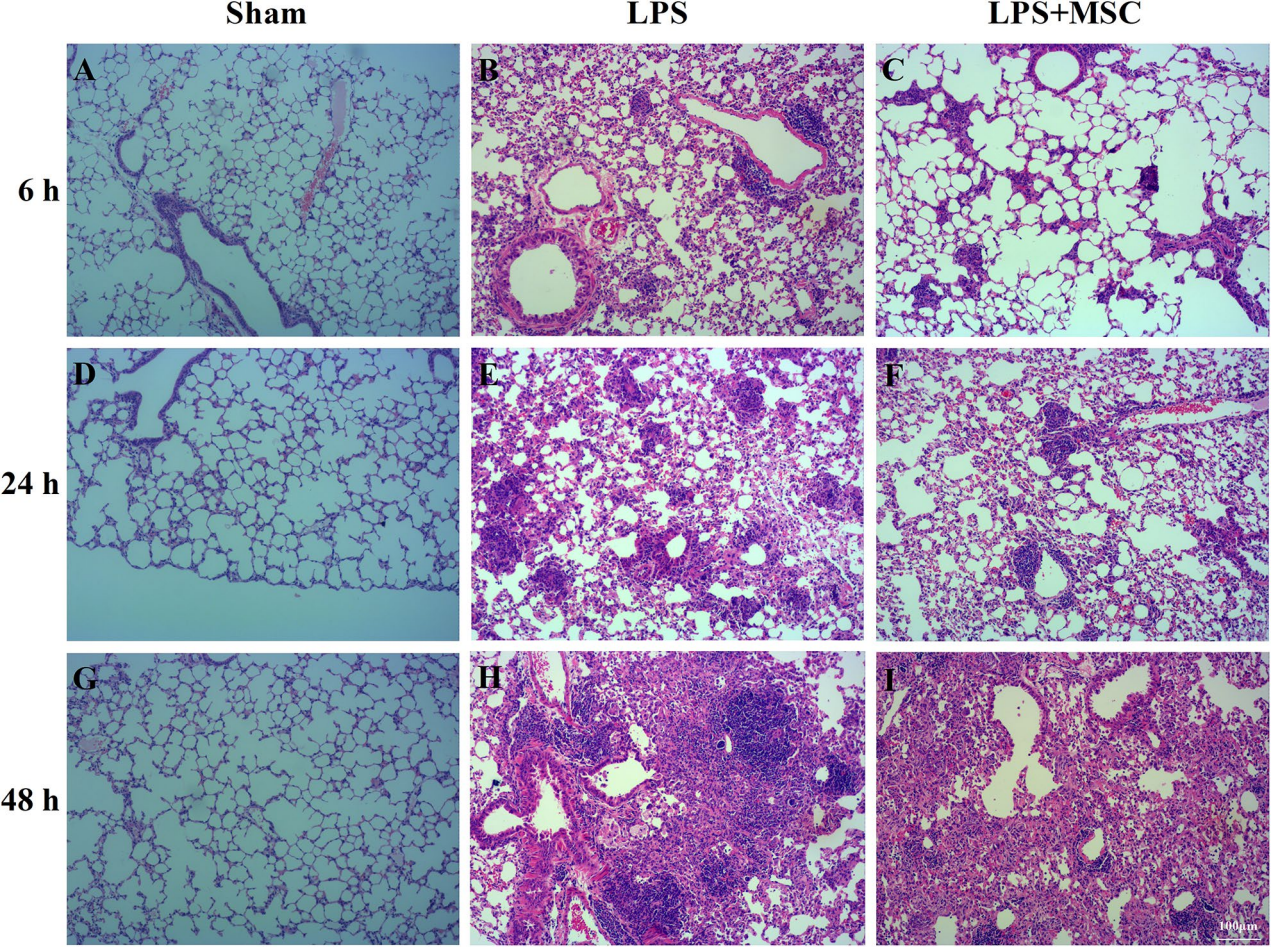
BM-MSCs administration reduces lung injury. Histological assessment of lung tissue from the three groups revealed the degree of lung injury, inflammatory cell infiltration, and interstitial edema. Compared with the lung tissue of normal mice (**A**, **D**, **G**), the degree of lung injury, inflammatory infiltration, and interstitial edema increased after LPS administration (**B**, **E**, **H**). Treatment with BM-MSCs significantly decreased the degree of lung injury, inflammatory cell infiltration, and interstitial edema (**C**, **F**, **I**) at each time point. (**A**–**L**, ×100 magnification).

Histological assessment showed evidence of inflammatory cell infiltration and interstitial edema. In the Sham group, there was no inflammatory cell infiltration and interstitial edema in lung tissue (Fig. 3A,D,G). The degree of lung injury, inflammatory infiltration, and interstitial edema increased after LPS administration (Fig. 3B,E,H). Compared with the LPS group, treatment with BM-MSCs prominently decreased the degree of lung injury, inflammatory cell infiltration, and interstitial edema (Fig. 3C,F,I). Thus, the histological analysis indicated that the administration of chicken BM-MSCs relieved the degree of lung injury at each time point.

### BM-MSCs attenuated neutrophil content in lung tissue

**Figure 4:**
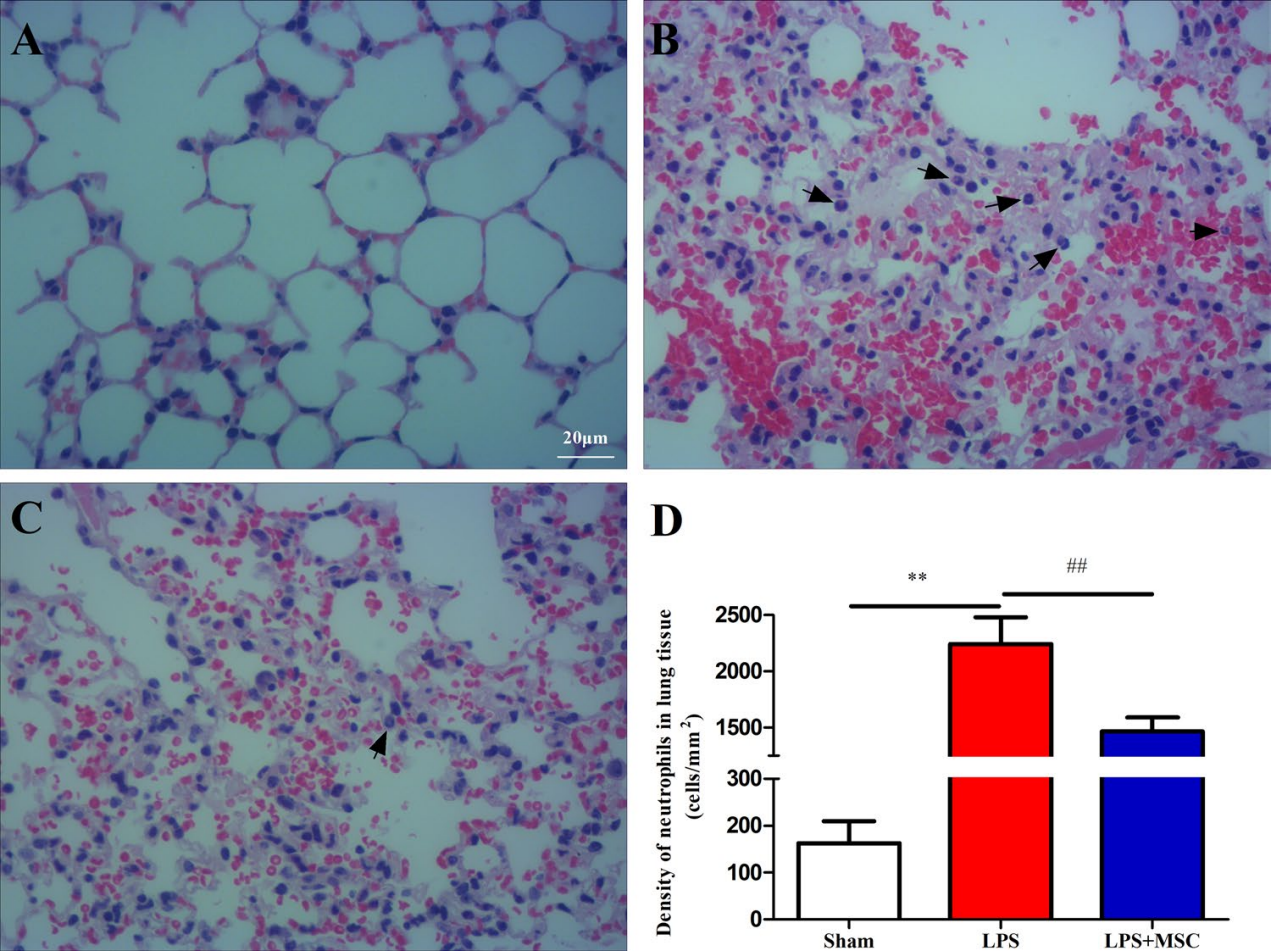
Neutrophil density in the lung tissue 6 h after LPS treatment. The microphotograph shows the density of neutrophils in the lung tissue. (**A**) Sham group, (**B**) LPS group, (**C**) LPS + MSC group, (**D**) the density of neutrophils in lung tissue. *P < 0.05 versus the Sham group; ^#^*P* < 0.05 versus the LPS group; **P < 0.01 versus the Sham group; ^##^*P* < 0.01 versus the LPS group. Values are expressed as the mean ± SD. Values are expressed as the mean ± SD. (**A**–**C**, ×100 magnification).

Micrographs were obtained to observe the density of neutrophils in the lung tissue. In the Sham group, there was no inflammatory cell infiltration in lung tissue (Fig. 4A). LPS injection prominently upregulated the density of neutrophils (black arrows) in lung tissue (Fig. 4B), while BM-MSCs administration prevented this increase in neutrophil content (Fig. 4C). Similarly, LPS administration prominently downregulated neutrophil content (*P* < 0.01). BM-MSCs administration prominently upregulated neutrophil content in lung tissue compared to LPS (Fig. 4D; *P* < 0.01).

As shown in Table 1, LPS injection prominently upregulated neutrophil content (*P* < 0.01) and macrophage content (*P* < 0.01) in BALF. BM-MSCs treatment prominently prevented the increase in neutrophil content (*P* < 0.01) and macrophage content (*P* < 0.01) compared to LPS. While LPS or BM-MSCs had no effect on lymphocyte count (*P* > 0.05) and eosinophil count (*P* > 0.05).

**Table 1:** Cell counts in the BALF (cells/mL) at 6 h after LPS treatment.

Group	Con	LPS	LPS + MSC
Total cells in BALF	1.35 ± 0.21	8.91 ± 2.14**	6.98 ± 0.94^##^
Neutrophils in BALF	0.04 ± 0.01	7.24 ± 1.67**	5.84 ± 2.38^##^
Lymphocytes in BALF	0.04 ± 0.01	0.15 ± 0.03	0.15 ± 0.04
Macrophages in BALF	1.07 ± 0.38	1.32 ± 0.27	0.96 ± 0.16
Eosinophils in BALF	0.02 ± 0.01	0.01 ± 0.01	0.04 ± 0.02

Increases in the numbers of total cells and neutrophils in the BALF were detected after LPS injection, while BM-MSCs prevented the increase in the numbers of total cells and neutrophils in the BALF. Treatment with LPS and BM-MSCs did not alter the numbers of lymphocytes, macrophages, or eosinophils in the BALF.

Values are expressed as the mean ± SD.

**P < 0.01 versus the Sham group.

^##^*P* < 0.01 versus the LPS group.

### BM-MSCs administration relieved pulmonary edema

**Figure 5:**
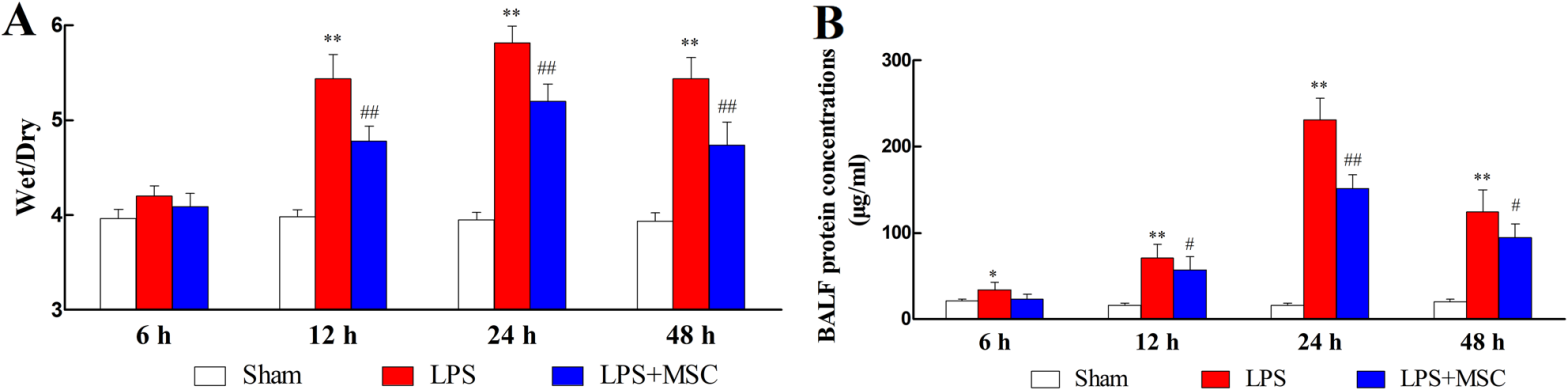
Effect of BM-MSCs on lung edema. (**A**) The wet/dry ratio of lung tissue. (**B**) Detection of total protein concentration. *P < 0.05 versus the Sham group; ^#^*P* < 0.05 versus the LPS group; **P < 0.01 versus the Sham group; ^##^*P* < 0.01 versus the LPS group. Values are expressed as the mean ± SD.

LPS injection prominently upregulated total protein concentration (Fig. 5A; *P* < 0.01) and W/D ratio (Fig. 5B; *P* < 0.01) compared to Sham. BM-MSCs treatment exerted a prominent preventative effect on the increases in total protein concentration (*P* < 0.05) and the W/D ratio (*P* < 0.05), demonstrating that BM-MSCs administration reduced the degree of pulmonary edema. As shown in Supplementary Material 4, LPS injection increased the Evans blue content in lung tissue compared to Sham (*P* < 0.01). BM-MSCs treatment attenuated the Evans blue content in lung tissue compared to LPS (*P* < 0.01).

### BM-MSCs administration reduced pulmonary inflammation

**Figure 6:**
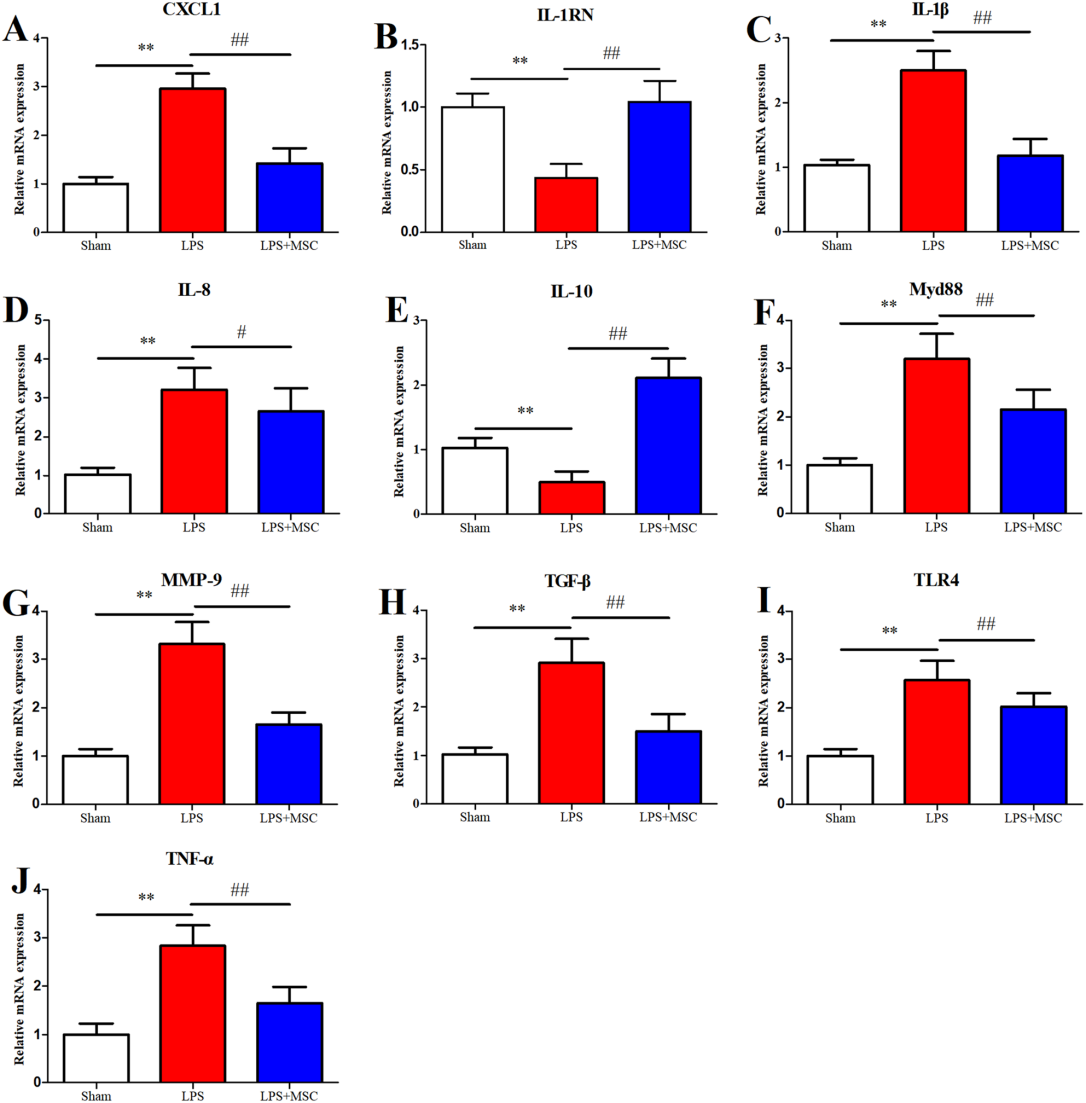
BM-MSCs prevented lung injury via the TLR4/Mdy88 pathway 6 h after LPS treatment. The gene expression levels of CXCL-1 (**A**), IL-1RN (**B**), IL-1β (**C**), IL-8 (**D**), IL-10 (**E**), Mdy88 (**F**), MMP-9 (**G**), TGF-β (**H**), TLR4 (**I**), and TNF-α (**J**) were detected. *P < 0.05 compared with the Sham group. *P < 0.05 versus the Sham group; ^#^*P* < 0.05 versus the LPS group; **P < 0.01 versus the Sham group; ^##^*P* < 0.01 versus the LPS group. Values are expressed as the mean ± SD.

LPS administration markedly increased CXCL-1 (Fig. 6A; *P* < 0.01), IL-1β (Fig. 6C; *P* < 0.01), IL-8 (Fig. 6D; *P* < 0.01), and TNF-α (Fig. 6J; *P* < 0.01) mRNA expression and markedly decreased IL-1RN (Fig. 6B; *P* < 0.01) and IL-10 (Fig. 6E; *P* < 0.01) mRNA expression in lung tissue. BM-MSCs administration markedly increased IL-1RN (*P* < 0.01) and IL-10 (*P* < 0.01) mRNA expression and markedly decreased CXCL-1 (*P* < 0.01), IL-1β (*P* < 0.01), IL-8 (*P* < 0.05), and TNF-α (*P* < 0.01) mRNA expression in lung tissue.

As shown in Table 2, LPS injection markedly upregulated BALF levels of CXCL-1 (*P* < 0.01), IL-6 (*P* < 0.01), IL-8 (*P* < 0.01), IL-10 (*P* < 0.01), and TNF-α (*P* < 0.01) and markedly downregulated BALF levels of IL-1RN (*P* < 0.01). BM-MSCs administration increased IL-10 (*P* < 0.01) and IL-1RN (*P* < 0.05) release and impaired CXCL-1 (*P* < 0.05), IL-6 (*P* < 0.05), IL-8 (*P* < 0.05), and TNF-α (*P* < 0.05) release in BALF compared to LPS.

**Table 2:** Cytokine levels in the BALF (pg/mL) at 6 h after LPS treatment.

	Con	LPS	LPS + MSC
CXCL-1	9.24 ± 1.65	594.82 ± 128.67**	466.4 ± 83.7^#^
IL-6	9.67 ± 2.33	1021.96 ± 297.69**	864.44 ± 130.75^#^
IL-8	12.38 ± 3.54	365.72 ± 82.66**	251.49 ± 76.51^#^
IL-1RN	54.15 ± 3.88	20.45 ± 10.79**	38.52 ± 15.17^#^
IL-10	9.62 ± 1.64	495.31 ± 136.6**	660.4 ± 174.1^##^
TNF-α	18.37 ± 16.38	343.82 ± 97.7**	211.45 ± 59.67^##^
MMP-9	12.64 ± 2.39	98.47 ± 11.54**	48.34 ± 6.73^##^
TGF-β	23.86 ± 5.29	132.98 ± 30.87**	103.41 ± 26.19^#^

Our data revealed that LPS increased the levels of CXCL-1, IL-6, IL-8, IL-10, MMP-9, TNF-α, and TGF-β and decreasing the levels of IL-1RN in the BALF. Compared with the LPS group, BM-MSCs administration increased the levels of IL-10 and IL-1RN and decreased the levels of CXCL-1, IL-6, IL-8, MMP-9, TNF-α, and TGF-β in BALF.

Values are expressed as the mean ± SD.

^#^*P* < 0.05 versus the LPS group.

**P < 0.01 versus the Sham group.

^##^*P* < 0.01 versus the LPS group.

### Effect of BM-MSCs on TLR4/Myd88 signaling pathway in lung tissue

LPS significantly activated Myd88 (Fig. 6F; *P* < 0.01) and TLR4 (Fig. 6I; *P* < 0.01) mRNA expression compared to Sham. BM-MSCs administration impaired LPS-induced Myd88 (*P* < 0.01) and TLR4 (*P* < 0.01) release.

### BM-MSCs administration reduced pulmonary fibrosis

LPS administration prominently upregulated profibrotic factors MMP-9 (Fig. 6. G; *P* < 0.01) and TGF-β (Fig. 6. H; *P* < 0.01) mRNA expression compared to Sham. While BM-MSCs administration prominently downregulated TGF-β (*P* < 0.01) and MMP-9 (*P* < 0.01) mRNA expression compared to the LPS group.

As Table 2 shows, LPS injection prominently upregulated BALF levels of profibrotic factors TGF-β (*P* < 0.01) and MMP-9 (*P* < 0.01) compared to Sham, while BM-MSCs administration prominently downregulated BALF levels of TGF-β (*P* < 0.05) and MMP-9 (*P* < 0.01) compared to LPS (*P* < 0.05).

**Figure 7:**
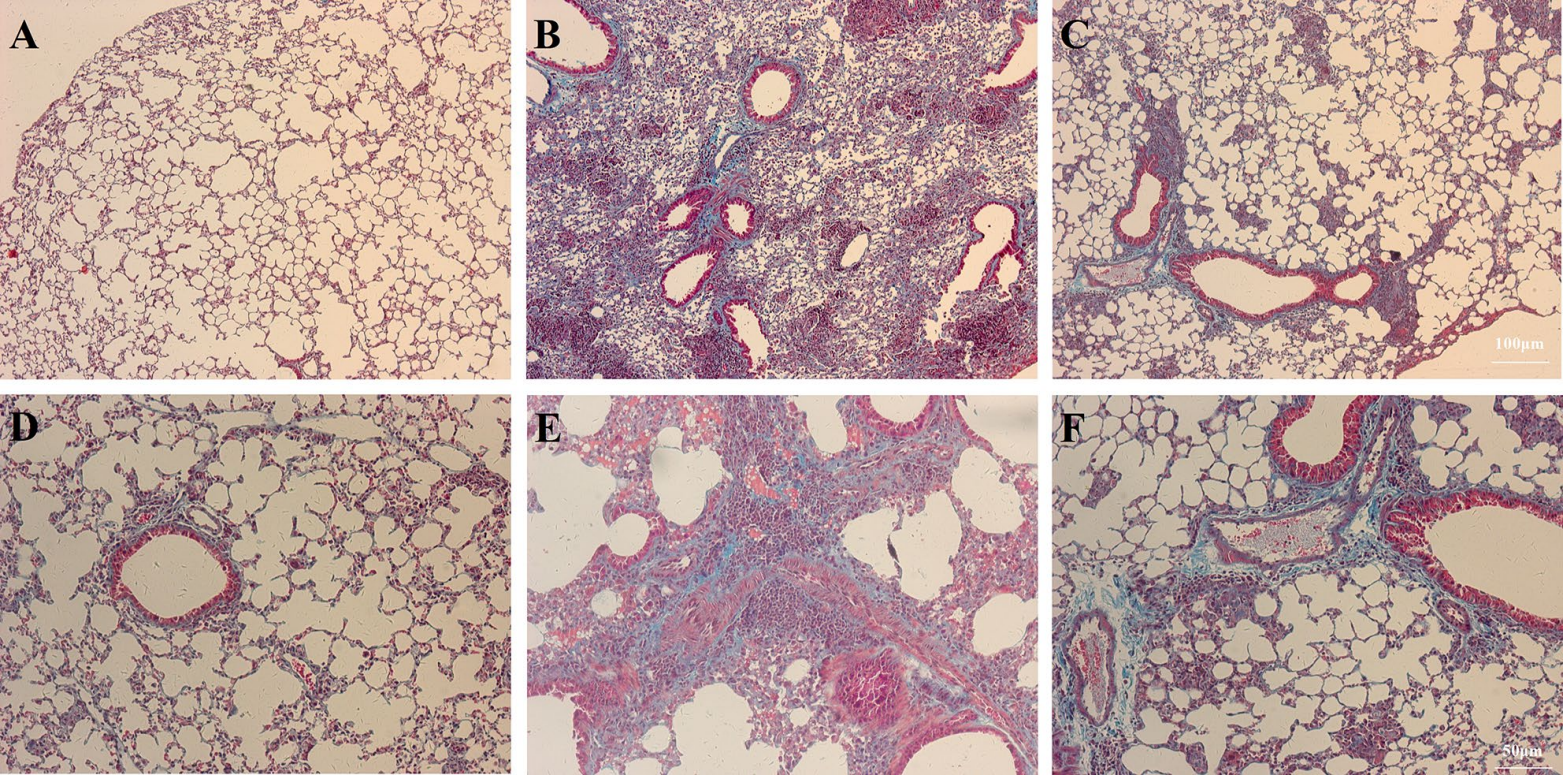
BM-MSCs improved LPS-induced pulmonary fibrosis at 24 h after LPS treatment. (**A**) Sham group. (**B**) LPS group. (**C**) LPS + MSC group. Mason staining revealed the degree of pulmonary fibrosis. (**A**–**C**, ×100 magnification. **D**–**F**, ×200 magnification).

Mason staining revealed the effects of BM-MSCs on pulmonary fibrosis. Compared with the Sham group (Fig. 7A,D), LPS injection prominently increased pulmonary fibrosis (Fig. 7B,E). While BM-MSCs treatment prominently inhibited the degree of pulmonary fibrosis (Fig. 7C,F) compared to LPS.

**Figure 8:**
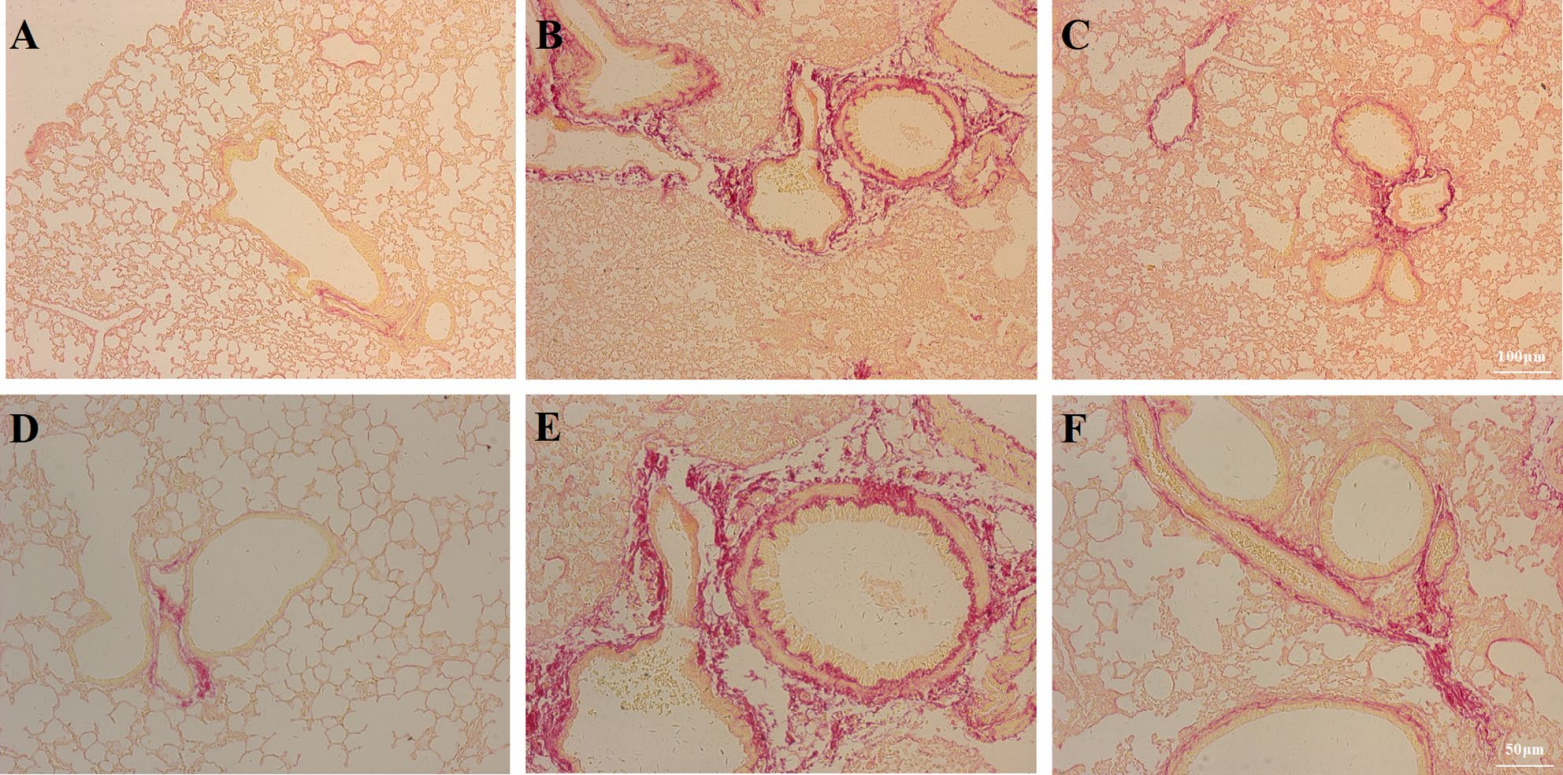
BM-MSCs improved LPS-induced pulmonary fibrosis in the airway wall at 24 h after LPS treatment. (**A**) Sham group. (**B**) LPS group. (**C**) LPS + MSC group. Sirius Red staining was performed to identify collagen fiber deposition in the airway wall. (**A**–**C**, ×100 magnification. **D**–**F**, ×200 magnification).

Sirius Red staining revealed the effects of BM-MSCs on pulmonary fibrosis in the airway wall. In the Sham group, there was no collagen fiber deposition in the airway wall (Fig. 8A,D). LPS injection resulted in increased collagen fiber deposition (Fig. 8B,E) compared to Sham. BM-MSCs administration resulted in decreased collagen fiber deposition compared to LPS (Fig. 8C,F).

### BM-MSCs attenuated LPS-induced extrapulmonary injury

#### BM-MSCs attenuated aortic injury

**Figure 9:**
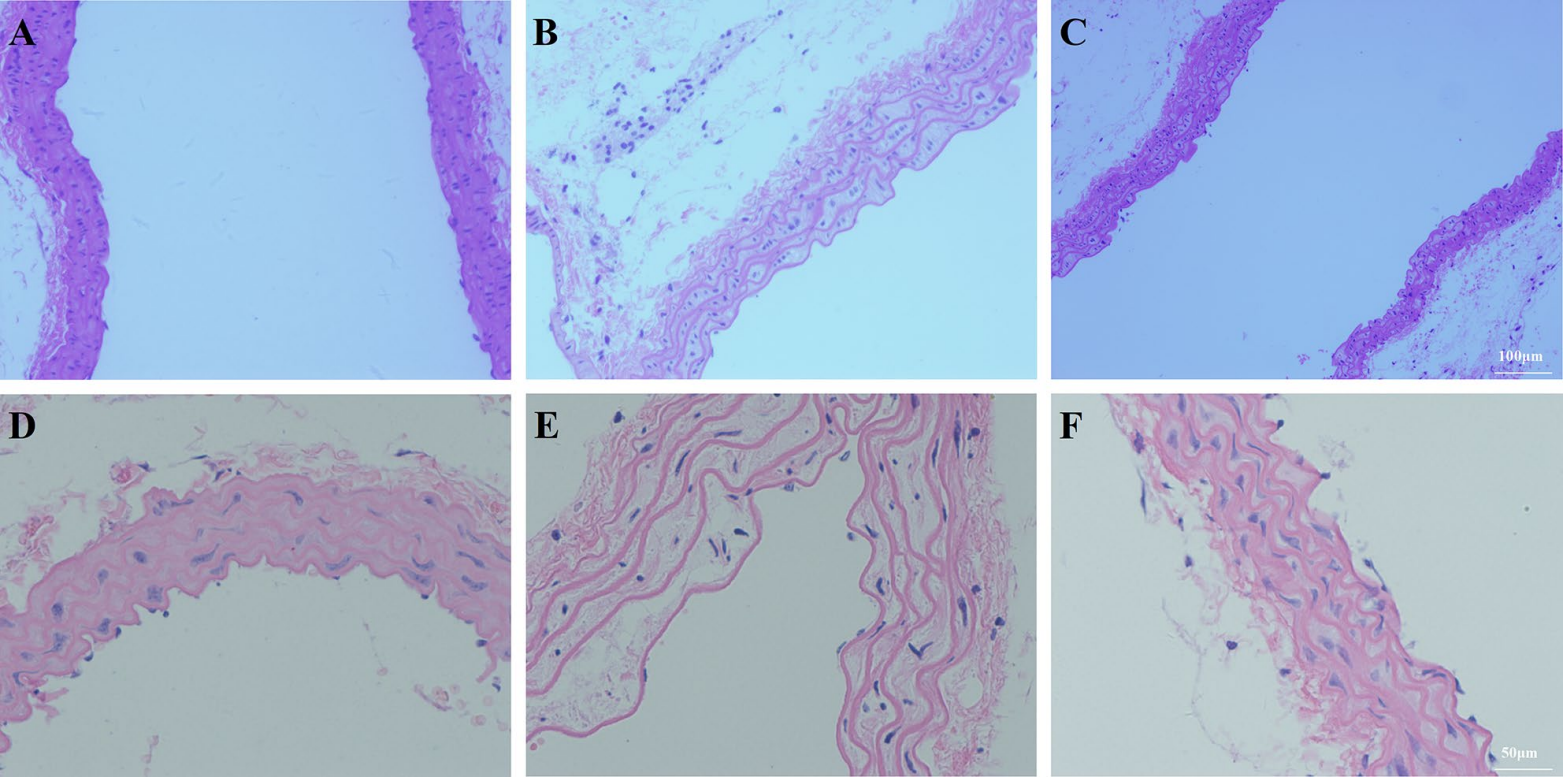
BM-MSCs improved LPS-induced aortic tissues at 48 h after LPS treatment. (**A**, **D**) Sham group. (**B**, **E**) LPS group. (**C**, **F**) LPS + MSC group. (**A**–**C**, ×100 magnification. **D**–**F**, ×200 magnification).

In the Sham group, the aorta was uniformly stained and arranged regularly, the endothelium was smooth and organized, and the elastic fibers had a regular, wavy shape (Fig. 9A,D). After LPS administration, the endothelium was not smooth or regular, the elastic fibers of the media became sparse, and the elastic fibers lost their regular, wavy shape (Fig. 9B,E). Compared with the LPS group, the shape of the endothelial elastic fibers and distribution of the elastic fibers were improved after BM-MSCs administration, but these fibers had not completely returned to their normal form (Fig. 9C,F).

As Table 3 shows, LPS administration increased the aortic intima media-thickness (*P* < 0.01) compared to Sham. BM-MSCs administration decreased the aortic intima media-thickness compared to LPS (*P* < 0.01).

**Table 3:** The degree of organ injury.

	Con	LPS	LPS + MSC
Alanine aminotransferase (U/L)	30.13 ± 7.51	148.33 ± 28.59**	114.27 ± 25.48^#^
Aspartate aminotransferase (U/L)	119.64 ± 14.96	180.67 ± 26.45**	150.25 ± 25.31^#^
Acute liver damage score	0.46 ± 0.06	3.57 ± 0.46**	2.16 ± 0.31^#^
Cre (μmol/L)	55.91 ± 11.98	214.39 ± 10.35**	184.47 ± 15.49^#^
BUN (mmol/L)	7.99 ± 1.64	13.56 ± 2.31**	10.59 ± 2.16^#^
Acute tubular damage score	0.34 ± 0.04	6.27 ± 0.85**	4.92 ± 0.61^#^
Medium membrane elastic fiber area ratio	48.93 ± 9.24	27.7 ± 4.01**	41.57 ± 8.34^##^

LPS injection increased the levels of the liver damage markers ALT and AST and the kidney damage markers Cre and BUN, which was improved by BM-MSCs administration. LPS administration increased the aortic intima media-thickness, which was reversed by BM-MSCs administration.

Values are expressed as the mean ± SD.

^#^*P* < 0.05 versus the LPS group.

**P < 0.01 versus the Sham group.

^##^*P* < 0.01 versus the LPS group.

#### BM-MSCs attenuated liver injury

**Figure 10:**
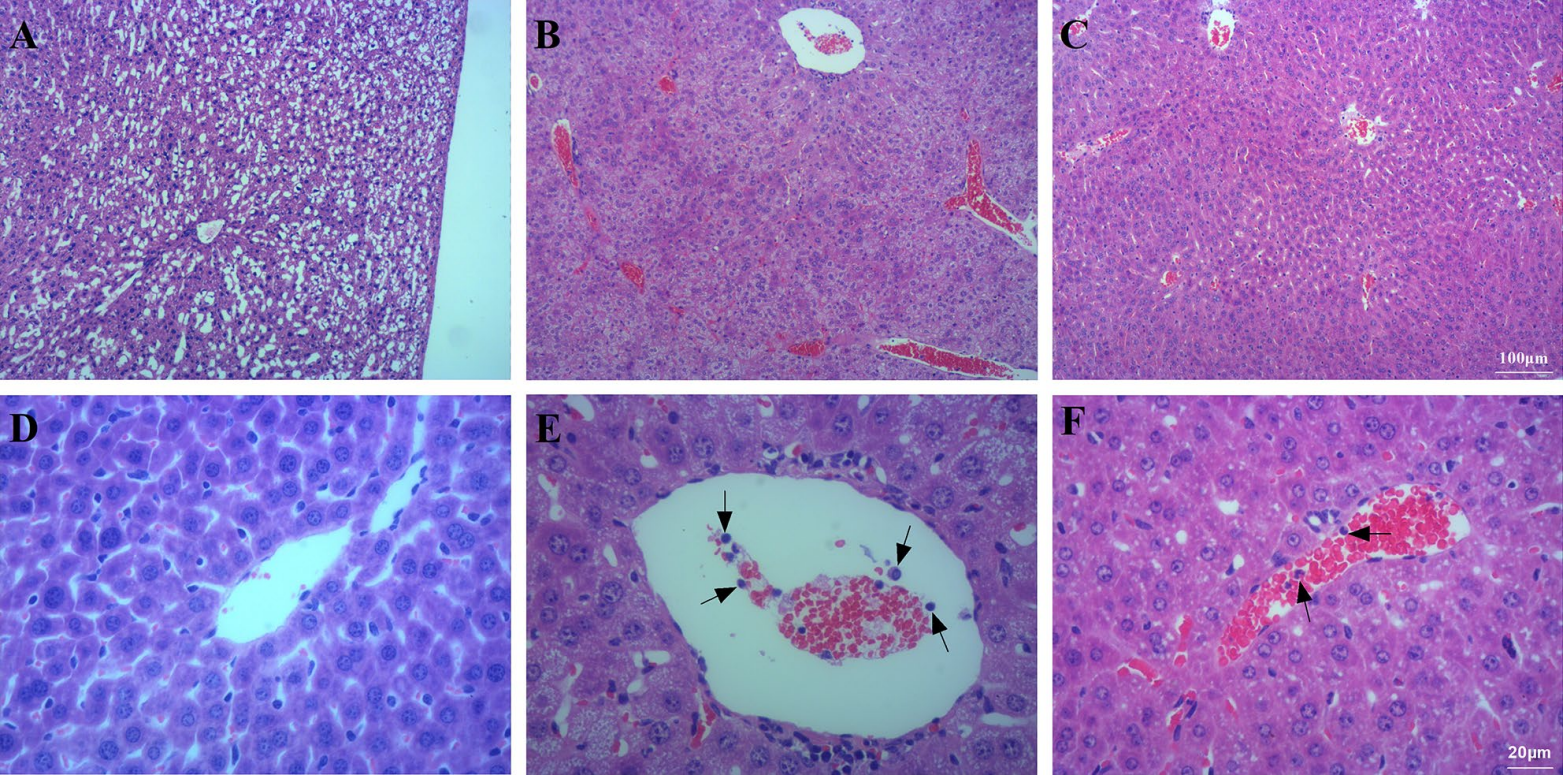
BM-MSCs improved LPS-induced liver injury at 48 h after LPS treatment. (**A**, **D**) Sham group. (**B**, **E**) LPS group. (**C**, **F**) LPS + MSC group. (**A**–**C**, ×100 magnification. **D**–**F**, ×400 magnification).

The liver lobules of mice in the Sham group (Fig. 10A,D) were intact and clear, the cells were neatly arranged, the intercellular space was free of edema, the liver structure was clear and regular, and no symptoms of injury were observed. The liver lobules of mice in the LPS group were severely damaged, the liver cells were swollen, the intercellular space was absent, and a large amount of neutrophil (black arrow) infiltration was observed (Fig. 10B,E). The liver lobules of mice in the LPS + MSC group showed significantly less structural damage to the liver tissue, with clearer liver lobules and infiltration of a small number of neutrophils (Fig. 10C,F).

As shown in Table 3, LPS administration increased the expression of ALT (*P* < 0.01) and AST (*P* < 0.01), which were markers of liver disease compared to Sham. BM-MSCs administration decreased the expression of ALT (*P* < 0.05) and AST (*P* < 0.05) compared to LPS.

#### BM-MSCs attenuated kidney injury

**Figure 11:**
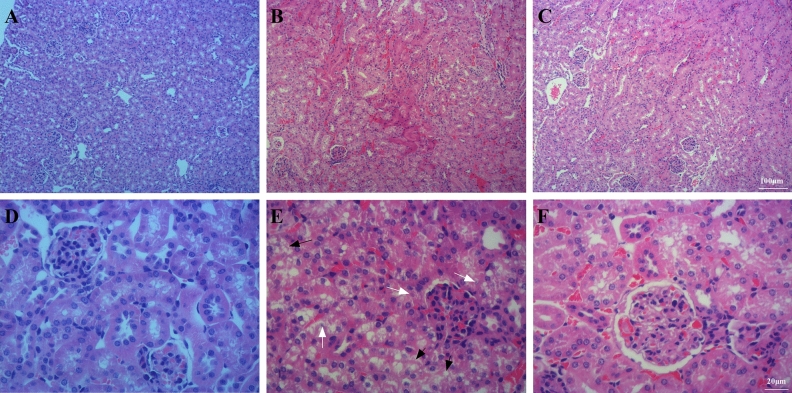
BM-MSCs improved LPS-induced kidney injury at 48 h after LPS treatment. (**A**) Sham group. (**B**) LPS group. (**C**) LPS + MSC group. (**A**–**C**, ×100 magnification. **D**–**F**, ×400 magnification).

The kidney tissues from mice in the Sham group were intact and clear, the cells were neatly arranged, the intercellular substance was free of edema, cortical tubular epithelial cells were well shaped, and almost every epithelial cell contained intact nuclei (Fig. 11A,D). The kidneys of mice in the LPS group were severely damaged (Fig. 11B,E), the cells were swollen, and the intercellular space was absent, accompanied by neutrophil infiltration and hemocytes. In addition, septic mice had severe epithelial vacuolization (black arrows) and some nuclei disappeared (white arrows). Compared with the LPS group, septic mice had significantly less kidney tissue structural damage, clearer nephrons, and less vacuolization after BM-MSCs administration (Fig. 11C,F), suggesting that BM-MSCs administration reduced LPS-induced kidney injury.

As shown in Table 3, compared with the Sham group, LPS administration increased levels of the kidney injury markers urea (*P* < 0.01) and BUN (*P* < 0.01), while urea (*P* < 0.05) and BUN (*P* < 0.05) levels were significantly decreased in the LPS + MSC group compared with those in the LPS group.

#### BM-MSCs attenuated heart injury

**Figure 12:**
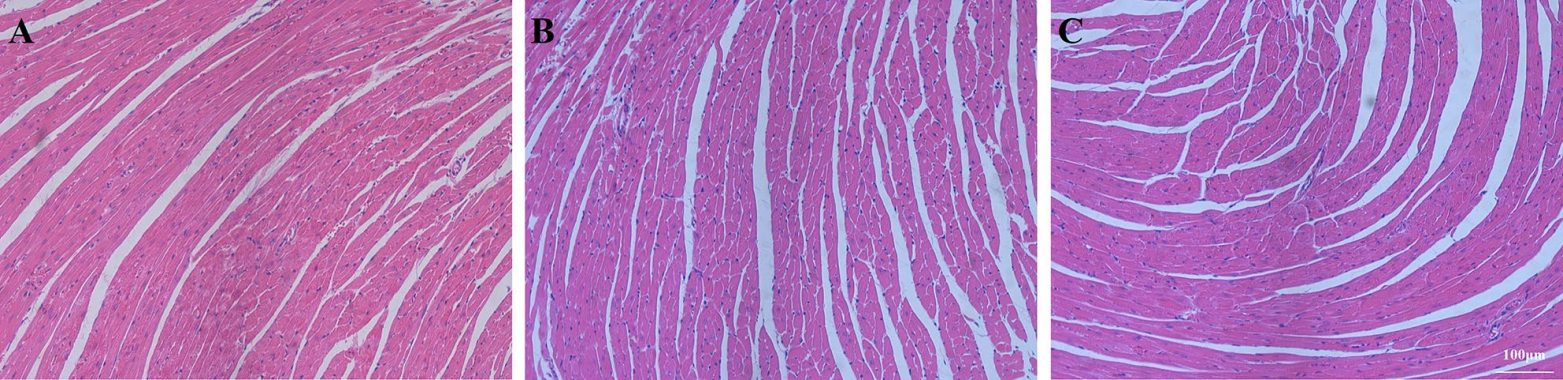
BM-MSCs improved LPS-induced heart injury at 48 h after LPS treatment. (**A**) Sham group. (**B**) LPS group. (**C**) LPS + MSC group. (**A**–**C**, ×100 magnification).

In the Sham group, the myocardial tissue was uniformly stained (Fig. 12A). In the LPS group, the myocardial tissue was disordered, myocardial degeneration and dissolution were observed, and a large number of inflammatory cells infiltrated the muscle space (Fig. 12B). The LPS + MSC group showed less inflammatory cell infiltration, and the myocardial fiber tissue structure was basically normal compared to the LPS group. The distribution of muscle fibers was improved but had not completely returned to its normal form (Fig. 12C).

### Detection of BM-MSCs homing in lung tissue

To obtain data indicating whether BM-MSCs migrate to the injured lungs, BM-MSCs were stained with CM-Dil, and the fluorescence signal was captured with a fluorescence microscope. DAPI staining was blue, and CM-Dil staining was green. Two weeks after LPS administration, engraftment of BM-MSCs was detected in the lung. Only a very small number of BM-MSCs were detected in the lung (Supplementary Material 5).

## Discussion

Here, we performed preliminary studies to solve some pivotal questions, such as simple and effective methods for the separation and culture of BM-MSCs. Next, we characterized BM-MSCs by evaluating their colony-forming and multidirectional differentiation capabilities and the levels of specific surface markers. Finally, we explored whether BM-MSCs administration exerts a positive protective effect against LPS-induced ALI, and potential mechanisms, including inflammation and oxidative stress, were studied.

Using bone marrow tissues obtained from 12-day-old chicken embryos, we separated and characterized a novel subset of MSCs, termed BM-MSCs, which showed unique mesenchymal stem cell-like properties. These plastic-adherent cells exhibited a spindle-like morphology similar to fibroblasts and possessed potent self-renewal potential. BM-MSCs derived from the embryonic mesoderm were induced to differentiate not only into cells that have an endoderm origin, such as adipocytes, osteoblasts, and chondrocytes but also into cells with an ectodermal origin, such as neuroblasts, confirming that BM-MSCs have cross-embryonic layer differentiation potential and further supporting their stem cell-like properties. The directional differentiation of the BM-MSCs was determined by the presence of different stimuli.

LPS administration leads to an excessive inflammatory response, pulmonary edema, and infiltration of inflammatory cells, which are the three main features of ALI^23,24^. Treatment with BM-MSCs improved the LPS-induced excessive inflammatory response, pulmonary edema, and inflammatory cell infiltration. Based on these results, BM-MSCs are a potential treatment for LPS-induced ALI.

An excessive inflammatory response is a crucial component of the pathogenesis of ALI. We identified that deleterious accumulation of neutrophils in lung tissue is a crucial component of ALI development, which is consistent with previous experimental results^25^. In our study, deleterious accumulation of neutrophils in the lung, liver, kidney, and heart tissues was detected after LPS administration. BM-MSCs administration inhibited deleterious accumulation of neutrophils in organ. Our data showed that BM-MSCs administration alleviated LPS-induced liver, kidney, and heart injury via suppressing LPS-induced neutrophil infiltration. The underlying molecular mechanisms through which BM-MSCs reduced the neutrophil content were unclear. Previous studies have shown that TNF-α, IL-8, and CXCL-1 were chemotactic factors for neutrophils^26,27^. Strikingly, our data demonstrated that BM-MSCs treatment impaired LPS-induced CXCL-1, IL-8, and TNF-α release in lung tissue. Therefore, BM-MSCs treatment inhibited neutrophil infiltration by impairing LPS-induced CXCL-1, IL-8, and TNF-α release.

MSCs were used to treat many autoimmune and inflammatory diseases, including sepsis and ALI owing to their paracrine action, low immunogenicity, and immunomodulatory effects^28,29^. Paracrine immunomodulation was one of the key mechanisms by which MSCs provided tissue (i.e., heart, lung) protection, which was tightly regulated by critical signaling pathways, including the PI3K/Akt, RAP1/NFκB, and MARPK/ERK pathways^30–32^. Some researches indicated that mitochondrial donation in MSC transplantation can alleviate lung injury^33,34^.

Paracrine immunomodulation was closely associated with paracrine action of MSCs, including TLR4/Myd88/NF-κB signaling^35^. Activation of TLR4/MyD88 signaling was a vital component in the development of ALI^36^. TLR4 was known as an important sensory receptor for LPS. The enhancement of TLR4 signaling pathway led to the up-regulation of inflammatory response^37^. Disruption of the TLR4 inhibited lung inflammatory response, confirming the role of TLR receptor signaling in inflammation-associated diseases^38,39^. TLR4/NF-κB signaling pathway enhanced the expression of inflammatory cytokine including IL-1β and TNF-α, which resulted in the inflammatory response aggravation. Blocking the expression of TLR4/NF-κB signal resulted in decreased inflammatory response^40,41^. Our data demonstrated that LPS administration prominently upregulated TRL4, Mdy88, TNF-α, and IL-1β mRNA in lung tissue. GMSCs administration prominently downregulated TRL4, Mdy88, TNF-α, and IL-1β mRNA expression during ALI. Similarly, GMSCs administration prominently downregulated BALF levels of TNF-α, and IL-1β during ALI. Hence, GMSCs administration impaired LPS-induced TNF-α and IL-1β release via inhibiting TRL4/Mdy88 expression. In addition, neutrophil accumulation in the lung, which was a characteristic feature of ALI, was attenuated in TLR4 mutant mice, indicating the involvement of TLR4 signaling in neutrophil inflammation in the lung^42,43^. Excessive neutrophil inflammation was a crucial component of the pathogenesis of ALI. Our data found that LPS administration led to deleterious accumulation of neutrophils in the lung^44,45^. Deleterious accumulation of neutrophils in the lung was prominently reduced after BM-MSCs administration. Hence, GMSCs may reduce LPS-induced neutrophil infiltration in the lung via suppressing TLR4/Myd88 signaling.

The oxidant/antioxidant balance was a vital component in the pathogenesis of ALI and that LPS administration led to oxidative stress injury^46^. In the present study, we found that BM-MSCs had an antioxidant effect. We demonstrated that BM-MSCs administration downregulated MDA and MPO expression but upregulated SOD and GSH expression in lung tissue. Hence, BM-MSCs alleviated ALI via modulating the oxidative/antioxidative balance.

Previous studies have ignored the effects of LPS and MSCs on the aorta. Generally, vascular injury occurs before the onset of pulmonary edema and inflammatory cell infiltration. Inflammatory cells, including neutrophils, attack blood vessels first, and the vascular injury results in increased lung permeability, inflammatory cell infiltration, and pulmonary edema.

Here, the protective effects of BM-MSCs on the aorta in acute lung injury mice were first demonstrated. We found that BM-MSCs administration prominently prevented the increase in the total protein concentration and W/D ratio. Based on these results, BM-MSCs protected against LPS-induced vascular injury.

Mason and Sirius Red staining showed the effects of BM-MSCs on pulmonary fibrosis. LPS injection prominently upregulated BALF levels of the profibrotic factors MMP-9 and TGF-β, which was reversed by BM-MSCs administration. Similarly, LPS administration prominently upregulated the mRNA expression levels of the MMP-9 and TGF-β compared to Sham, while BM-MSCs administration prominently downregulated the mRNA expression levels of TGF-β and MMP-9 compared to the LPS group. This evidence showed that BM-MSCs administration alleviated pulmonary fibrosis via impairing LPS-induced TGF-β and MMP-9 release.

In contrast to traditional therapeutic methods, MSC-based therapy was a comprehensive intervention treatment. Previous studies have mainly focused on single factors and single pathways. The idea that one or two pathways exert a decisive role in ALI was not comprehensive and may explain why traditional methods for treating ALI are ineffective. However, MSC administration exerted a positive effect on ALI through multiple mechanisms simultaneously, including inhibition of an excessive inflammatory response and oxidative stress injury. In addition, BM-MSCs administration decreased vascular injury and lung edema. All of these factors combined improved the survival rate of ALI mice. Overall, chicken BM-MSCs serve as a potential alternative resource for stem cell therapy and exert a prominent effect on organ injury including lung, liver, kidney, heart, and aortic injury. BM-MSCs administration increased the antioxidant and anti-inflammatory capacities during ALI.

BM-MSCs reduced the excessive and uncontrolled inflammatory response involving inflammatory mediators and effector cells, among which neutrophils play a key role. BM-MSCs reduced the neutrophil content, in part because BM-MSCs inhibited the expression of CXCL-1, IL-8, and TNF-α. BM-MSCs altered the levels of inflammatory mediators, partially through inhibition of TLR4/Myd88 signaling.

Previous work demonstrated that different tissue origin-derived MSC displayed different expression levels of human leukocyte antigen (HLA) responsive to inflammatory stimuli^47–51^. Some studies suggested that MSCs had low immunogenicity, while other studies showed that MSCs were not immunogenic. We suggested that MSCs exhibited low immunogenicity. Hence mice were intraperitoneally injected with the anti-rejection drug cyclosporine for one week before the experiment. Compared with bone marrow- and adipose-derived MSC, GMSCs may display different expression levels of HLA responsive to inflammatory stimuli. Our data did not detect the expression levels of HLA responsive to LPS administration.

### Limitations

Some limitations of our research should not be ignored. Our data demonstrated that BM-MSCs have multidirectional differentiation capabilities. However, the mechanisms underlying the differentiation of BM-MSCs are unclear. Hence, further research into the inducers and differentiation mechanisms is desperately needed, and these studies will be quite important for cell-based treatment and regenerative medicine. MSCs play a prominent role in both restoration of ALI and in protecting vascular injury and restoring endothelial barrier function. Generally, vascular injury occurs before the onset of pulmonary edema and inflammatory infiltration. Our data demonstrated that BM-MSCs protected against aortic injury. However, the underlying molecular mechanisms involved in the preventive effect of MSCs in ALI-associated aortic injury have not yet been fully elucidated. Our data ignored the effects of BM-MSCs on the change in lung function index and pulmonary hypertension.

## Supplementary Information

Supplementary Information 1.

Supplementary Information 2.

Supplementary Information 3.

Supplementary Information 4.

Supplementary Information 5.

Supplementary Information 6.
