# Supplementary material for: Chicken bone marrow mesenchymal stem cells improve lung and distal organ injury
Source: Sci Rep. 2021 Sep 10;11:17937. doi: 10.1038/s41598-021-97383-4 (PMC8433226; doi:10.1038/s41598-021-97383-4)
Supplement: Supplementary file 1 — Supplementary Information 1. [file 41598_2021_97383_MOESM1_ESM.docx]

**Supplementary material 1.** Composition of the medium used to culture chicken BM-MSCs.

| Complete medium | DMEM/F12, 10% FBS, 5 ng/mLbFGF, 2 mM L-glutamine |
| --- | --- |
| Adipocyte induction medium | DMEM/F12, 10% FBS, 1 mM dexamethasone, 0.5 mM IBMX, 10 mg/mLinsulin and 60 mM indomethacin |
| Osteogenic induction medium | DMEM/F12, 10% FBS, 0.5 mM dexamethasone, 10 mM β-glycerophosphate and 50 mg/mLvitamin C |
| Chondrogenic induction medium | DMEM/F12, 10% FBS, 1% ITS, 50 mg/mLL-proline, 0.1 mM dexamethasone, 0.9 mM sodium pyruvate, 50 mg/mLvitamin C and 10 ng/mLTGF-β3 |
| Neurogenic inducting medium | DMEM/F12, 2% B27, 1% glutamine, 40 ng/mLmedium-1 bFGF, 20 ng/mL EGF, 1 μM all-trans-retinoic acid, and 100 μM 2-mercapoethanol |
