# Supplementary material for: Chicken bone marrow mesenchymal stem cells improve lung and distal organ injury
Source: Sci Rep. 2021 Sep 10;11:17937. doi: 10.1038/s41598-021-97383-4 (PMC8433226; doi:10.1038/s41598-021-97383-4)
Supplement: Supplementary file 2 — Supplementary Information 2. [file 41598_2021_97383_MOESM2_ESM.docx]

**Supplementary material 2.** The primer sequences used for BM-MSC identification.

| Gene name | Primer sequence |
| --- | --- |
| CXCL1 | AAGGATGGTCGCAATG |
|  | GGTGGCTAAGTCTGAGGT |
| IL-1β | CAGAACAGCCGGACTTTC |
|  | CTTGCTCGCCTTCACCAC |
| IL-6 | AGGGACCCAAAGGAGACAT |
|  | GCACGGAAATTCCTGTTGAT |
| IL-8 | GCCATCTGCTACACAGGTGA |
|  | AAAGGCTCCTCAGGTTCTGG |
| IL-10 | ACCGCCTTGTCGTTAGACTG |
|  | GAATCTCCATCGTCCTCCAC |
| IL-1RN | GCTCATTGCTGGGTACTTACAA |
|  | CCAGACTTGGCACAAGACAGG |
| TLR4 | ATGGCATGGCTTACACCACC |
|  | GAGGCCAATTTTGTCTCCACA |
| Myd88 | TCATGTTCTCCATACCCTTGGT |
|  | AAACTGCGAGTGGGGTCAG |
| MMP-9 | CTGGACAGCCAGACACTAAAG |
|  | CTCGCGGCAAGTCTTCAGAG |
| GAPDH | CACTGTCCACGCCATCACT |
|  | CCTGTTGCTGTAGCCGAATT |
