# Supplementary material for: Chicken bone marrow mesenchymal stem cells improve lung and distal organ injury
Source: Sci Rep. 2021 Sep 10;11:17937. doi: 10.1038/s41598-021-97383-4 (PMC8433226; doi:10.1038/s41598-021-97383-4)
Supplement: Supplementary file 3 — Supplementary Information 3. [file 41598_2021_97383_MOESM3_ESM.docx]

**Supplementary material 3.** The results of flow cytometry.


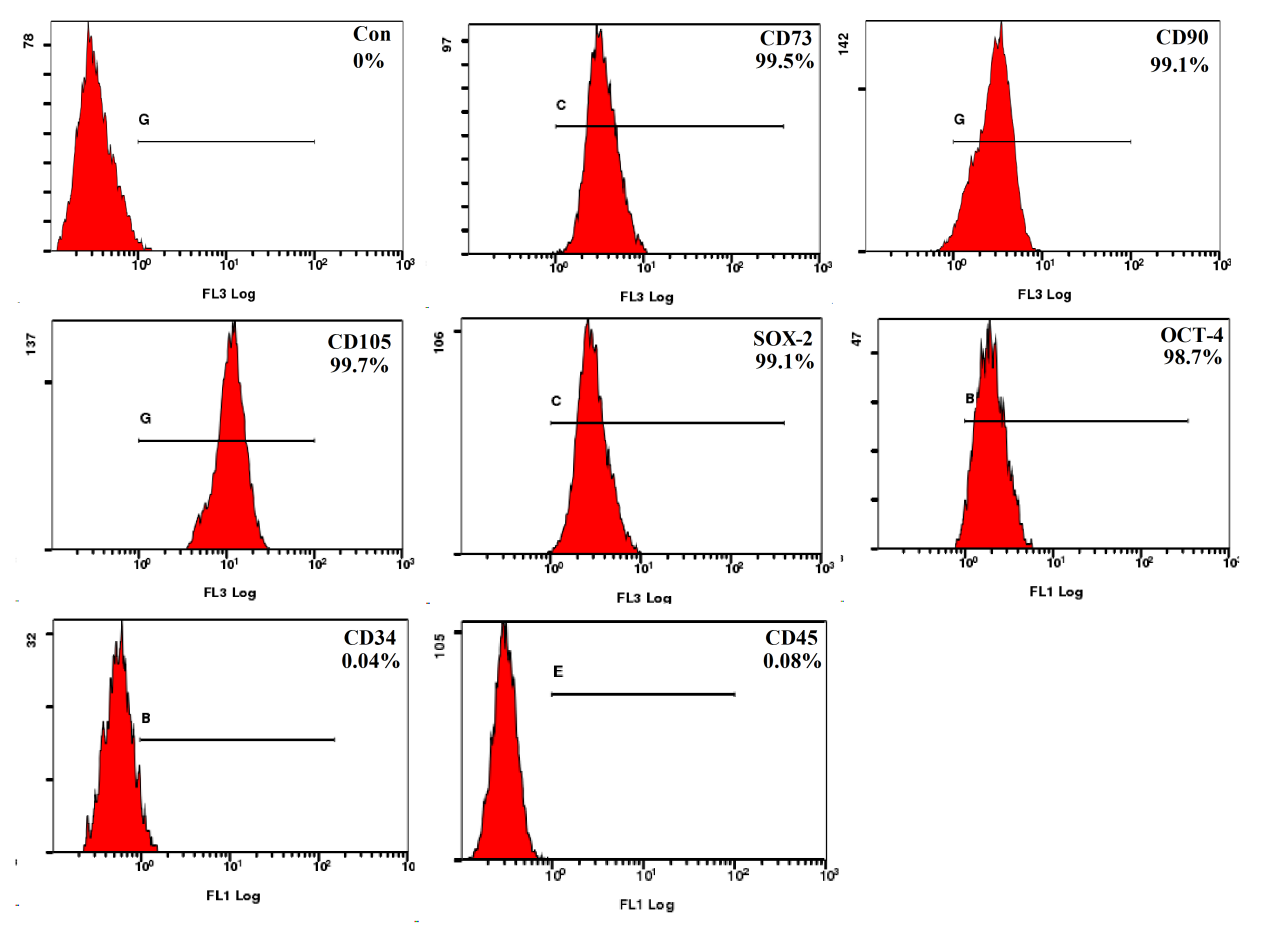


The BM-MSCs surface antigens CD73, CD90, CD105, Sox-2, and OCT-4 were positively expressed, while CD34 and CD45 were negatively expressed, as identified by flow cytometry.
