# Supplementary material for: Chicken bone marrow mesenchymal stem cells improve lung and distal organ injury
Source: Sci Rep. 2021 Sep 10;11:17937. doi: 10.1038/s41598-021-97383-4 (PMC8433226; doi:10.1038/s41598-021-97383-4)
Supplement: Supplementary file 4 — Supplementary Information 4. [file 41598_2021_97383_MOESM4_ESM.docx]

**Supplementary material 4.** The results of Evans blue leakage test.


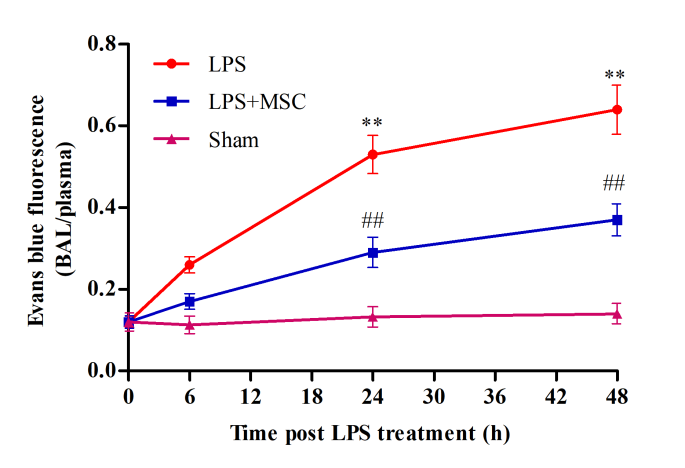


LPS injection increased the Evans blue content compared to Sham, while BM-MSCs administration attenuated Evans blue content compared to the LPS group. ^*^, *P* < 0.05 versus the Sham group; ^#^ *P* < 0.05 versus the LPS group; ^**^ *P* < 0.01 versus the Sham group; ^##^ *P* < 0.01 versus the LPS group. Values are expressed as the mean ± SD.
