# Supplementary material for: Chicken bone marrow mesenchymal stem cells improve lung and distal organ injury
Source: Sci Rep. 2021 Sep 10;11:17937. doi: 10.1038/s41598-021-97383-4 (PMC8433226; doi:10.1038/s41598-021-97383-4)
Supplement: Supplementary file 5 — Supplementary Information 5. [file 41598_2021_97383_MOESM5_ESM.docx]

**Supplementary material 5.** BM-MSCs homing in lung tissue.


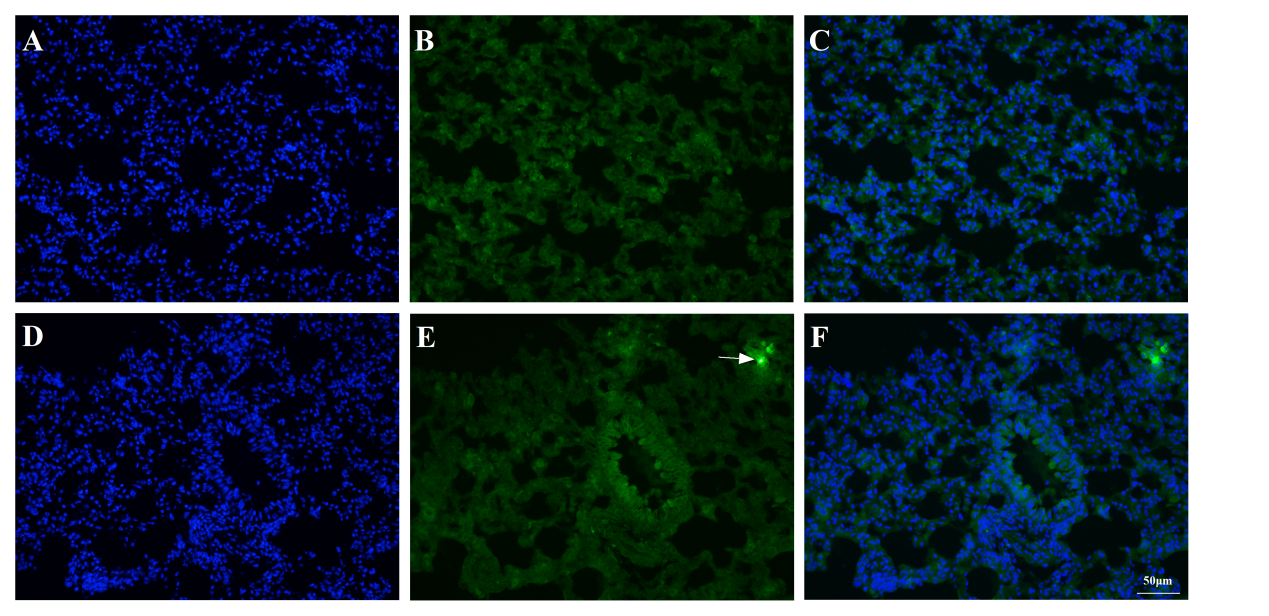


The CM-Dil-labeled BM-MSCs in the lung were detected. **(A, D)** DAPI, **(B, E)** fluorescence, **(C, F)** Merge. A, B, and C = LPS group. D, E, and F = LPS +MSC group. White arrow, CM-Dil-labeled BM-MSCs. Two weeks after the BM-MSCs injection, only a very small percentage of the MSCs were successfully localized in lung tissue. (**A-F, 200x** magnification).
