# Supplementary material for: Chicken bone marrow mesenchymal stem cells improve lung and distal organ injury
Source: Sci Rep. 2021 Sep 10;11:17937. doi: 10.1038/s41598-021-97383-4 (PMC8433226; doi:10.1038/s41598-021-97383-4)
Supplement: Supplementary file 6 — Supplementary Information 6. [file 41598_2021_97383_MOESM6_ESM.docx]

**Supplementary material 6.** Detection of neutrophil content, lung injury score, kidney injury score, liver injury score, and pulmonary fibrosis score.

**Neutrophil content:** By using a 100-point grid of a known area (62,500 μm^2^ at 400X magnification) that was attached to the ocular of the microscope, we counted the number of points that were hitting the alveolar tissue and the numbers of neutrophils and positive cells in each field. The cell density was determined as the number of positive cells in each field divided by the tissue area^1^. Morphometric measurements were performed in 15 fields for each animal at 400X magnification by an investigator who was blinded to the specific group that was studied.

**Kidney injury score:** Acute tubular damage was assessed via hematoxylin and eosin (HE) staining by using previously described criteria^2^. Acute tubular damage was scored by using a semiquantitative scoring system (0 to 3 points) for each criterion in 20 randomly sampled high-power fields of the cortex per animal (0 = absent, 1 = mild, 2 = moderate, and 3 = strong acute tubular damage).

**Liver injury score:** Formalin-fixed liver tissues were embedded in paraffin wax, serially sectioned, and then stained with HE. Morphological characteristics (including PMN infiltration, interstitial edema, focal necrosis, and hemorrhage/congestion) were evaluated under a light microscope, and Suzuki scores (i.e., the sum of the scores of congestion [0: none; 4: severe], vacuolization [0: none, 4: severe], and necrosis [0: none; 4: > 60%]) were calculated to determine the liver injury level^3,4^.

**Lung injury score:** The lung tissues were exposed to paraffin procedures and sectioned at approximately 5 μm thick, after which they were stained with HE, as has been previously described. Lung injury was evaluated and scored by two pathologists who were blinded to the experimental design by using a recent criterion^5^, in which lung damage was evaluated on a two-point scale with scores ranging from 0 to 1.

**Myocardial injury score:** Cardiac sections were prepared as previously described and stained with HE according to standard procedures. A pathologist was assigned to grade the MI injuries in a blinded fashion. Histological analyses of infarct size, hemorrhage, and leukocyte infiltration were scored as being none, weak, moderate, strong, or very strong (scores of 0, 1, 2, 3, or 4, respectively). This method for the objective quantification of MI injury has been previously described ^6-8^.

**Detection of pulmonary fibrosis:** To assess alveolitis and fibrosis, Masson-stained sections were evaluated via semiquantitative histology by a pathologist who was blinded to the treatment groups, with the pathologist using a light microscope and a scoring system for the measurements, as has been previously described^9,10^. A histological semiquantitative examination of the lung was performed on sections after standard Sirius Red staining. Periportal fibrosis was staged 0-4, and perisinusoidal fibrosis was scored 0-2, thus giving a maximum possible score of 6^11^.


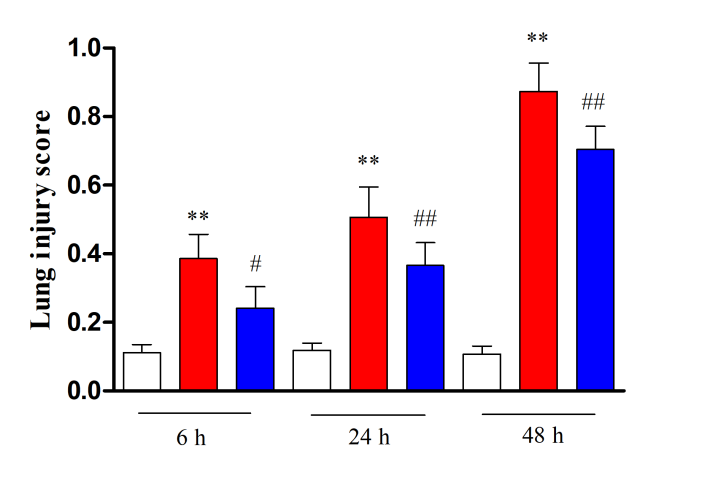


**Lung injury scores**. LPS injection increased lung liver injury score, acute tubular damage score, and myocardial injury score, which was improved by GMSCs administration. White bars = Sham group, Red bars = LPS group, Blue bars = LPS +MSC group. ^*^, *P* < 0.05 versus the Sham group; ^#^, *P* < 0.05 versus the LPS group; ^**^, *P* < 0.01 versus the Sham group; ^##^, *P* < 0.01 versus the LPS group. Values are expressed as the mean ± SD.

**Organ injury score**

| **Group** | **Con** | **LPS** | **LPS + MSC** |
| --- | --- | --- | --- |
| Liver injury Score | 1.61 ± 2.33 | 4.96 ± 2.69^**^ | 3.47 ± 0.47^#^ |
| Acute tubular damage score | 1.43 ±0.17 | 5.73 ± 0.66^**^ | 3.42 ± 0.41^#^ |
| Myocardial injury score | 1.15 ± 0.18 | 4.49 ± 0.59^**^ | 3.52 ± 0.57^#^ |

LPS injection increased liver injury score, acute tubular damage score, and myocardial injury score, which was improved by BM-MSCs administration. ^*^, *P* < 0.05 versus the Sham group; ^#^, *P* < 0.05 versus the LPS group; ^**^, *P* < 0.01 versus the Sham group; ^##^, *P* < 0.01 versus the LPS group. Values are expressed as the mean ± SD.

**Reference**

[1] Lanças T, Kasahara DI, Prado CM, et al. Comparison of early and late responses to antigen of sensitized guinea pig parenchymal lung strips. J Appl Physiol. 2006;100:1610-1616. doi: 10.1152/ japplphysiol.00828.2005.

[2] Bockmeyer CL, Reuken PA, Simon TP, et al. ADAMTS13 activity is decreased in a septic porcine model. Significance for glomerular thrombus deposition. Thromb Haemost 2011;105:145-153. doi: 10.1160/TH10-03-0153.

[3] Cay-Huyen Chen, Pei-Shan Tsai, Chun-Jen Huang. Minocycline ameliorates lung and liver dysfunction in a rodent model of hemorrhagic shock/resuscitation plus abdominal compartment syndrome. J Surg Res. 2013;180(2):301-9. doi: 10.1016/j. jss.2012.04.036.

[4] S Suzuki, S Nakamura, T Koizumi, et al. The beneficial effect of a prostaglandin I2 analog on ischemic rat liver. Transplantation. 1991;52(6):979-83. doi: 10.1097/ 00007890-199112000-00008.

[5] Gustavo Matute-Bello, Gregory Downey, Bethany B Moore, et al. An official American Thoracic Society workshop report: features and measurements of experimental acute lung injury in animals. Am J Respir Cell Mol Biol. 2011;44(5):725-38. doi: 10.1165/rcmb.2009-0210ST.

[6] Paul-Mihai Boarescu , Ioana Chirilă, Adriana E Bulboacă, et al. Effects of Curcumin Nanoparticles in Isoproterenol-Induced Myocardial Infarction. Oxid Med Cell Longev. 2019;2019:7847142. doi: 10.1155/2019/7847142.

[7] Erman Caner Bulut, Leyla Abueid, Feriha Ercan, et al. Treatment with oestrogen-receptor agonists or oxytocin in conjunction with exercise protects against myocardial infarction in ovariectomized rats. Exp Physiol. 2016;101(5):612-27. doi: 10.1113/EP085708.

[8] Mohamed Abdellah Ibrahim, Ayman Geddawy, Soha Abdel-Wahab, et al. Sitagliptin prevents isoproterenol-induced myocardial infarction in rats by modulating nitric oxide synthase enzymes. Eur J Pharmacol. 2018;829:63-69. doi: 10.1016/ j.ejphar. 2018.04.005.

[9] Szapiel SV, Elson NA, Fulmer JD, et al. Bleomycin-induced interstitial pulmonary disease in the nude, athymic mouse. Am Rev Respir Dis. 1979;120:893‑899. doi: 10.1164/arrd.1979.120.4.893.

[10] Fulmer JD, Bienkowski RS, Cowan MJ, et al. Collagen concentration and rates of synthesis in idiopathic pulmonary fibrosis. Am Rev Respir Dis. 1980;122: 289-301. doi: 10.1164/arrd.1981.124.3.341a.

[11] Sebastian Huss, Jörg Schmitz, Diane Goltz, et al. Development and evaluation of an open source Delphi-based software for morphometric quantification of liver fibrosis. Fibrogenesis Tissue Repair. 2010; 17;3(1):10. doi: 10.1186/1755-1536-3-10.
